# Snoezelen in people with intellectual disability or dementia: A systematic review

**DOI:** 10.1016/j.ijnsa.2023.100152

**Published:** 2023-08-25

**Authors:** Gemma Testerink, Annet ten Brug, Gerdine Douma, Annette van der Putten

**Affiliations:** a Academic Collaborative Centre for Profound Intellectual and Multiple Disabilities, Department of Pedagogical and Educational Sciences, Faculty of Behavioural and Social Sciences, University of Groningen, Grote Rozenstraat 38, 9712 TJ Groningen, The Netherlands; b 's Heeren Loo, Berkenweg 11, 3818 LA Amersfoort, The Netherlands

**Keywords:** Snoezelen, Multisensory environment, Intellectual disability, Dementia

## Abstract

**Background:**

Snoezelen focuses on multisensory stimulation in an adapted environment and was originally developed for people with severe and profound intellectual (and multiple) disabilities. Snoezelen has been used for many years with various target groups and for different purposes. Variation in its application has resulted in a lack of understanding of snoezelen's application characteristics and of how they may relate to effects.

**Objective:**

The aim of this review was to provide an overview of the application and effects of snoezelen in people with intellectual disability or dementia in order to analyse the relationship between application characteristics and effects.

**Design:**

A systematic review.

**Methods:**

Five databases were searched for snoezelen studies that took place in a specially adapted environment. The methodological quality of the included studies was assessed using the Mixed Methods Appraisal Tool. The application characteristics (that is, the stimuli used, environment, and support given) and the effects were extracted. Reported effects were categorized into different human functioning dimensions using the model of intellectual disabilities of the American Association on Intellectual and Developmental Disabilities.

**Results:**

In total, 62 studies involving people with intellectual disability (*n* = 30) or dementia (*n* = 32) were included. An overview of snoezelen used in other target groups (*n* = 24) is provided as supplementary material. Details on the application of snoezelen were often lacking. A total of 10 application characteristics (for example, frequency, role of the support person) were extracted. All studies reported the presence of a support person (*n* = 62; 100%). Effects were found in all five human functioning dimensions. The most-reported effects (61.3% overall) related to mental health, such as a reduction in challenging behaviour and improved mood. In a minority of studies (*n* = 10, 16.1%), effects on the support person were also reported. Due to limited details about the application of snoezelen and the large variation in measured effects, analysing the relationship between these was impossible.

**Conclusions:**

The majority of studies lacked details on application characteristics during snoezelen. Reported effects varied, although most related to mental health. Future research should analyse in detail the relationship between application and effects.

## 1 Contribution of the paper

**What is already known about the topic?**

• Snoezelen has been used in practice with various target groups and in various ways for decades.
• Although positive effects have been reported, the relationship between application and effects is still unclear, which limits specific applications.

**What this paper adds**

• Application characteristics (regarding approach, application, context, and conditions) are more concrete and can be considered in the use of snoezelen.
• We provide insight into a range of reported effects of snoezelen in people with intellectual disabilities or dementia; most related to mental health
• Readers will gain a preliminary understanding of the working mechanisms of snoezelen.

## 2 Background

Snoezelen focuses on experiencing sensory stimuli, such as auditory, visual, tactile, olfactory, and gustatory stimuli, in an adapted environment and, if needed, with a support person (Hulsegge and Verheul, 1987). Snoezelen is used with varying effects in a range of target groups worldwide (Cameron et al., 2020; Hogg et al., 2001; Lancioni et al., 2002). Evaluating the use and effect of snoezelen in the original target group, namely people with severe or profound intellectual (and multiple) disabilities, is challenging due to limited research (Vlaskamp and Nakken, 2008). However, broadening this perspective and analysing the use of snoezelen in other target groups may provide an insight into working mechanisms that explain the relationship between application and effects. The scope of this review, therefore, consisted of snoezelen and the use of multisensory environments in line with, or evolved from, Hulsegge and Verheul's (1987) original description.

The term ‘snoezelen’ is made up of two Dutch words: ‘snuffelen’ (sniffing) and ‘doezelen’ (dozing). ‘Sniffing’ can be interpreted as an active way of exploring sensory stimuli and ‘dozing’ as a more relaxing way of experiencing these same stimuli. The original aim of snoezelen was to find a balance between relaxation and activation (Hulsegge and Verheul, 1987). Snoezelen was specifically developed in the late 1970s for people with severe or profound intellectual (and multiple) disabilities. The premise was that people with severe or profound intellectual (and multiple) disabilities primarily explore, experience, and understand the world through the senses, often have a limited ability to explore their surroundings by themselves, and may easily be overwhelmed by everyday stimuli (Hulsegge and Verheul, 1987). Snoezelen was originally developed to offer a suitable activity to experience joy, explore, and learn in an inviting environment (Hulsegge and Verheul, 1987). There was no substantiated theoretical framework nor a strict intervention guide for how to apply snoezelen. However, the support persons applying snoezelen, often nurses or activity providers, were provided with several basic principles: creating the right ambiance, allowing users to choose and to set the pace, ensuring the right duration, using repetition, and providing stimuli in a selective manner, the right basic attitude of support persons, and the right support (Hulsegge and Verheul, 1987). These original principles highlight the role of the support person as enabler in the application of snoezelen. Hulsegge and Verheul (1987) stressed that in order to apply these elements ‘correctly’, they had to be individually shaped to a person's needs. Because snoezelen appealed to many healthcare professionals and parents/relatives, it was widely used in the Netherlands and abroad (Hulsegge and Verheul, 1987; Vlaskamp and Nakken, 2008). Nowadays, snoezelen is used for recreational, therapeutic, and educational purposes (Cameron et al., 2020; Hogg et al., 2001). In addition, the effect of snoezelen on the quality of working life of support persons has also attracted interest (Collier et al., 2018; Van Weert, Van Dulmen, Spreeuwenberg, Bensing, and Ribbe, 2005; Zhang et al., 2020).

Initially snoezelen was used in target groups particularly at risk of understimulation, such as people with intellectual disabilities or dementia. The use of snoezelen was based on the assumption that sensory stimulation provides a meaningful activity for people, especially those with limited cognitive abilities (S. W. C. Chan et al., 2010; Silva et al., 2018). Being able to engage in a sensory activity supposedly has a positive effect on physical and mental wellbeing because it counteracts sensory deprivation (Silva et al., 2018).These target groups were at risk not just of understimulation but also of overstimulation. In this regard, snoezelen mostly relied on the assumption that snoezelen induces relaxation. Induced relaxation is assumed to result in various positive outcomes, such as reduced challenging behaviour and improved adaptive behaviour (S. W. C. Chan et al., 2010). Later, snoezelen was also applied in other target groups that were particularly at risk of overstimulation and who would benefit from reduced arousal levels and stress regulation (Cameron et al., 2020; Haig and Hallett, 2023; Ismail et al., 2021).

Much is still unclear about how providing sensory stimulation, adjusted to the individual's sensory needs, influences effects. This is occasionally explained in terms of Ayres's (1979) sensory integration theory, which focuses on the influence of sensory stimuli on arousal levels (Haigh and Mytton, 2016; Novakovic et al., 2019). There is limited knowledge about the extent to which other factors, such as social contact, contribute to the effects of snoezelen (Cameron et al., 2020). In light of evidence-based practice, a substantiated use of snoezelen and knowledge about its effects became increasingly important (Hogg et al., 2001; Vlaskamp and Nakken, 2008). The purposeful use of snoezelen is hampered by a lack of understanding of its working mechanisms (Cameron et al., 2020).

Literature reviews have sought to evaluate the effectiveness of snoezelen in people with intellectual disability (Botts et al., 2008; Breslin et al., 2020; S. W. C. Chan et al., 2010; Hogg et al., 2001; Lotan and Gold, 2009), people with dementia (Chung et al., 2010; Hayden et al., 2022; Sánchez et al., 2013; Silva et al., 2018), mental health problems or stress related issues (Haig and Hallett, 2023; Ismail et al., 2021), and multiple target groups (Cameron et al., 2020; Lancioni et al., 2002). Although the results of these reviews are often inconclusive, the overall conclusion is that snoezelen can have positive effects on these various target groups (for example, S. W. C. Chan et al., 2010; Haig and Hallett, 2023; Lotan and Gold, 2009; Silva et al., 2018) and their support persons (Cameron et al., 2020). However, these reviews do not consider the application of snoezelen in relation to the observed effects (Cameron et al., 2020; Hogg et al., 2001). Therefore, we still do not know how application characteristics can be used to achieve specific snoezelen outcomes (Cameron et al., 2020). In addition to the existing knowledge, this review aims firstly to present a current overview of the application characteristics and effects of snoezelen for both snoezel attendees and their direct support persons. Secondly, it aims to analyse the relationship between application characteristics and effects. A greater understanding of the working mechanisms of snoezelen will help theory building on how to use snoezelen, for what purpose, and with whom. In addition, it may provide information for support persons about how they can put snoezelen into practice, depending on the person and the purpose of the activity.

## 3 Methods

### 3.1 Eligibility criteria

A systematic review was conducted of studies written in English, published in peer-reviewed academic journals between 1985 and 12 April 2023, and with full text available. Articles that studied the effect of snoezelen on participants or support persons were included, while non-empirical studies (for example, reviews and meta-analyses) were excluded. Qualitative studies, either open (such as interviews) or semi-open (such as surveys) were included if they evaluated perceived benefits of snoezelen. Articles were excluded if they did not focus on an overall research question (for example, case studies illustrating the use of snoezelen). Snoezelen had to take place in an adapted multisensory environment; this could also be referred to as multisensory stimulation, multisensory intervention, or multisensory therapy. Studies aimed at multisensory stimulation through reminiscence-like activities were excluded.

### 3.2 Search and selection strategy

The search was conducted in the following databases: Medline, PsycINFO, ERIC, CINAHL, and Embase. The original search date was 24 December 2019, and the search was updated 12 April 2023. The search strategy and limits were similar in all databases; the search strategy in Medline is presented in Table 1 as an example. The search resulted in 969 unique articles, of which 200 titles and abstracts were first screened independently by two researchers (author 1 and peer 1) with 96% consensus. Screening of the remaining articles was done by one researcher (author 1). Next, two researchers (author 1 and author 2) independently appraised the eligibility of 17% of the available full texts. At first, there was 68% agreement, and full consensus was reached after fine-tuning the criteria (for example, appraising case studies). One researcher (author 1) conducted a further appraisal of the eligibility of full texts. A snowball method was applied to the references in excluded reviews and meta-analyses on snoezelen and included articles.

**Table 1:** Search string and search limits in medline.

Search string in Medline	Database limits
Snoezelen OR multisensory stimulation OR multisensory stimulation OR multisensory intervention OR multisensory intervention OR multisensory therapy OR multisensory therapy OR multisensory environment OR multisensory environment	1985–12 April 2023 English language Peer-reviewed academic journals Human

The search began broadly, with no target group limitation. Two main groups emerged from the search, namely people with intellectual disabilities or dementia. Other target groups were too various in nature to be substantially clustered based on diagnoses. Also, given the original description of snoezelen involving intervention in both understimulation and overstimulation, we proceeded with the two most represented target groups at risk of both. Subsequently, data collection on the ‘other’ target groups was limited to an overview of these studies, which is available as supplementary material; see S-Table I.

### 3.3 Data collection

Data was collected independently by one researcher (author 1) and checked by the research team (that is, authors 2 and 4). Items included research design, participant characteristics, characteristics of the main elements of snoezelen, and reported effects of snoezelen on participants or support persons. We reported an effect if it was indicated as such in the particular study. Established and presumed relationships between participants, application characteristics, and effects were also extracted.

### 3.4 Assessment of methodological quality

A quality assessment of the studies was performed using the Mixed Methods Appraisal Tool (Pluye and Hong, 2014). This critical appraisal tool consists of two screening questions, followed by five criteria specific to quantitative, qualitative, or mixed-method designs. For optimal study quality, all five questions had to be answered affirmatively (that is, ‘yes’, the item was properly addressed in the study, as opposed to ‘can't tell’ or ‘no’). To summarize the quality appraisal we scored the number of times a ‘yes’ was mentioned, with a maximum score of 5 out of 5. A low- quality appraisal was often due to omitted details rather than reported items lowering the quality of the research. Based on these factors, we decided not to exclude articles on the basis of the quality appraisal. Supplementary material was made available to provide additional details on the origin of the findings.

First, 10 articles were independently appraised by two researchers (author 1 and author 2) for practice purposes and to fine-tune the criteria. Second, a further 10 studies were independently appraised, resulting in 100% agreement on the screening questions and design category. The agreement on design- specific criteria was 55%. Lack of consensus was due to different interpretations of a few items; namely, whether participants were representative of the target group, whether groups were comparable at baseline, and whether participants adhered to the intervention. Both authors agreed on how these items should be scored. One researcher (author 1) completed the remaining quality assessments and consulted the second researcher (author 2) when in doubt.

### 3.5 Data synthesis

Qualitative analysis was used to identify characteristics of snoezelen regarding the application of sensory stimuli, the multisensory environment, and the support person. Where applicable, characteristics were grouped; for example, room size and blockage of daylight were grouped under physical aspects of the multisensory environment. We calculated the number of studies that reported a characteristic. Reported effects were categorized into human functioning dimensions. Because snoezelen was originally developed for people with severe or profound intellectual (and multiple) disabilities, we used the multidimensional model of intellectual disability of the American Association on Intellectual and Developmental Disabilities, which includes the following dimensions: 1) intellectual functioning, 2) adaptive behaviour, 3) health, 4) participation, and 5) context (Schalock et al., 2021). An overview was created in which the application characteristics and outcomes were shown in relation to each other. We analysed whether there were shared participant or application characteristics in the studies, with and without effects. The calculated percentages refer to the number of studies in the target group; if no specific target group was addressed, the percentage refers to the total number of studies.

## 4 Results

### 4.1 Study selection

**Fig. 1:**
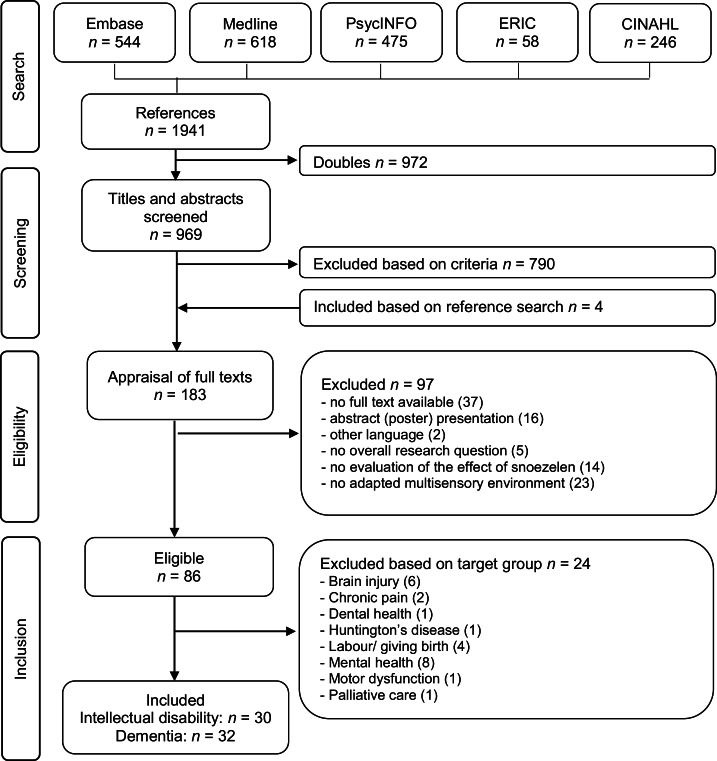
Flowchart selection process. Note: *n* = number of studies.

In total, 62 articles were included (see Fig. 1). In 30 studies, the participants were people with intellectual disabilities and, in 32 studies, people with dementia. An overview of studies with ‘other’ target groups (*n* = 24) is provided as supplementary material (S-Table I). The study characteristics, results, and quality appraisal are summarized for each included article in Table 2.

**Table 2:** Overview of study characteristics, results, and quality appraisal of studies on snoezelen.

#	Author (↓), year Country	Design Control condition	Quality appraisal (5/5 = maximum score)	Participants 1) *n* (age) 2) female/male 3) level of ID/DM 4) setting	Study aim	Snoezelen 1) goal 2) individual/group 3) frequency 4) duration 5) approach/strategies used	Data collection	Results
01	Anderson et al., 2011 Australia	Mixed method Control: garden	Qualitative: 4/5 Non-randomized: 1/5 Mixed: 1/5	1) *n* = 12 (*M* = 89 yrs; *SD* 8.19; range 81–94) 2) NR 3) moderate to severe DM 4) residential	Evaluate the impact of multisensory therapy on behaviours and engagement	1) to calm distressed people 2) individually 3) 1x per week 4) 20 min 5) the participant was invited to sit in a recliner chair and engaged using stimuli provided	Observation of behaviour using time- sampling coding 4 categories (disturbed/ disengaged, neutral, engaged, very engaged) Focus group with staff	No effect on engagement Perceived benefits: ↑ relationship between staff and participants
02	Ayer, 1998 United Kingdom	Qualitative study	Failed screening criteria	1) *n* = NR staff 2) NR 3) NA (staff); snoezelen used with participants with profound multiple ID 4) 1 day centre, 2 schools	Explore the use of MSEs and describe the experiences of participants	NA	Semi-structured questionnaires completed by staff	Perceived benefits: ↓ challenging behaviour; ↑ mood, relaxation, and interaction with social and physical environment
03	Baillon et al., 2004 United Kingdom	RCT; crossover Control: reminiscence	1/5	1) *n* = 20 (*M* = 73.5 yrs) 2) 12F, 8 M 3) mild to severe DM 4) residential and day centre	Compare the effects of snoezelen and reminiscence therapy on agitated behaviour	1) NR 2) individually 3) 3x in 2 weeks 4) 40 min 5) structure depended on the participant; the content was in accordance with non-specified guidelines	Agitation Behaviour Mapping Instrument; Interact scale; heart rate	Effect, but no difference from control condition: ↓ agitation, ↑ mood, interaction
04	Baillon et al., 2005 United Kingdom	RCT; crossover Control: reminiscence	1/5	1) *n* = 20 (*M* = 73.5 yrs) 2) 12F, 8 M 3) mild to severe DM 4) residential and day centre	Assess the effects of snoezelen on agitated behaviour	1) NR 2) individually 3) 3x in 2 weeks 4) 40 min 5) structure depended on the participant; the content was in accordance with non-specified guidelines. Participant's preferences were used.	Agitation Behaviour Mapping Instrument; Interact Short scale; heart rate	(see Baillon et al., 2004)
05	Baker et al., 2001 United Kingdom	RCT Control: activity sessions	2/5	1) *n* = 50 (*M* = 78 yrs; all but one over 60) 2) 25F, 25 M 3) moderate to severe DM 4) day centre	Evaluate the immediate effects of multisensory stimulation and the carry-over of effects on behaviour and mood	1) NR 2) individually 3) 2x per week 4) 30 min 5) non-directive and enabling approach; efforts to stimulate all senses except taste; unpatterned, nonsequential stimuli; no intellectual/physical demands; guideline for internal structure; positive attitude of staff	Interact; Interact Short; Rehabilitation Evaluation Hall and Baker Tool; Behaviour and Mood Disturbance Scale; Behaviour Rating Scale; Mini-Mental State Examination; Cognitive Assessment Scale	Effect, but no difference from control condition: ↑ mood, interaction Effects carried over to the home environment for multisensory stimulation, not for activity sessions No long-term effects
06	Baker et al., 2003 United Kingdom, Netherlands, Sweden	RCT Control: activity sessions	1/5	1) *n* = 136 (experimental group: *M* = 81 yrs) 2) NR 3) moderate to severe DM 4) residential and day centre	Assess whether multisensory stimulation is more effective in changing the behaviour, mood and cognition than activity sessions	1) provide atmosphere of trust, warmth and confidence 2) individually 3) 2x per week 4) 30 min 5) non-directive approach; following participant's lead; emphasis on all senses except taste; no intellectual or physical demands; unpatterned and non-sequential stimuli; internal session structure; preferences were investigated beforehand; equipment was introduced slowly, one item at a time	Interact; Interact Short; Behaviour observation scale for intramural psycho-geriatrics; Mini-Mental State Examination; Behaviour Rating Scale; Behaviour and Mood Disturbance Scale; Rehabilitation Evaluation Hall and Baker Tool	No effect on cognitive status, mood, interaction Slight improvements were reported for both groups No long-term effects
07	Bauer et al., 2012 Australia	Quantitative descriptive study; cross-sectional study	Failed selection criteria	1) *n* = 416 facilities 2) NA 3) dementia 4) residential	Build a comprehensive picture of the use of multisensory interventions for the management of dementia- related behaviours	NA	Computer-assisted telephone interview	Perceived benefits: 17% of the participants believed multisensory interventions (including snoezelen) had a positive benefit in the management of dementia-related behaviours
08	Bauer et al., 2015 Australia	Non-randomized study Control: common best practice	1/5	1) *n* = 16 (snoezelen group: *M* = 81 yrs; range 70–99) 2) 11F, 5 M 3) moderate to severe DM 4) residential	Evaluate the impact of snoezelen on wandering and restlessness compared to common best practice	1) NR 2) NR 3) 2x per week 4) NR 5) implemented based on the support person's knowledge of the resident and prior experience	Behavioural observation chart on wandering and restlessness in 4 categories. (behaviour stopped or person settled, behaviour improved, behaviour ongoing, behaviour worsened)	Effect, but no difference from control condition: ↓ wandering and restlessness
09	Berkheimer et al., 2017 United States of America	Non-randomized study; crossover Control: exercise programme	2/5	1) *n* = 8 (*M* = 88 yrs; range 80–95) 2) 6F, 2 M 3) dementia 4) residential	Compare the effects of a snoezelen programme and an exercise programme on agitation	1) NR 2) up to 2 participants 3) 1x per week 4) 30 min 5) support person supervises participant's activity and encourages engagement	Cohen-Mansfield Agitation Inventory short-form	No effect on anxiety Slight decrease in anxiety was reported for both groups
10	Carter and Stephenson, 2012 Australia	Quantitative descriptive study; cross-sectional study	3/5	1) *n* = 19 schools 2) NR 3) NA (staff); snoezelen used in severe ID 4) school	Study the use of MSEs	NA	Questionnaires completed by staff	Perceived benefits (most-reported benefits): ↓ challenging behaviour, anxiety; ↑ sensory stimulation, relaxation, focus, mood, motivation to learn, interaction with environment, relationship with staff
11	Chan and Chien, 2017 Hong Kong	RCT Control: usual care, massage therapy, and MSE and massage therapy combined	3/5	1) *n* = 42 (*M* = 43.40 yrs; *SD* 10.92; range 18–64) 2) 25F, 17 M 3) severe to profound ID, additional diagnoses include cerebral palsy, epilepsy, hearing loss, and visual impairment. 4) residential	Evaluate the effectiveness of MSE and massage therapy, either separately or combined, in reducing challenging behaviours	MSE alone condition: 1) NR 2) NR 3) 2x per week 4) 30 min 5) enabling approach; participants could choose preferred equipment; they were encouraged to play and interact. *The combined massage therapy and MSE group received 20* min *massage therapy during 30* min *MSE.*	Behaviour Problem Inventory; pulse and respiration rates; Alertness Observation Checklist; Behaviour Checklist	Effect, no difference from combined massage therapy and MSE: ↑ relaxation No effect on challenging behaviour
12	Chan et al., 2005 Hong Kong	RCT Control: activity sessions	2/5	1) *n* = 89 (11 ≥ 71) 2) 53F, 36 M 3) mild to severe ID, additional diagnoses include schizophrenia/ psychosis, behavioural disorders, and personality disorders. 4) residential	Evaluate the impact of multisensory therapy on pulse rate, relaxation, challenging, stereotypic self-stimulating, and adaptive behaviours	1) NR 2) group of 5 or 6 3) 3x per week 4) 60 min 5) non-directive enabling approach; avoiding interactions	Behavioural Relaxation Scale; pulse rate; Snoezelen Diary Card; Checklist of Challenging Behaviour; Behaviour Checklist	Effect compared to control: ↑ mood, relaxation No effect on adaptive skills and challenging behaviour
13	Chan et al., 2007 Hong Kong	RCT Control: activity sessions	0/5	1) *n* = 89 (11 ≥ 71) 2) 53F, 36 M 3) mild to severe ID, additional diagnoses include schizophrenia/ psychosis, behavioural disorders, and personality disorders. 4) residential	Evaluate the efficacy of multisensory therapy in moderating behaviour and to understand perceived benefits and difficulties in the implementation of multisensory therapy	1) NR 2) group of 5 or 6 3) 3x per week 4) 60 min 5) no interfering or correcting; prompting and encouraging participants to explore the environment and touch objects of choice	Behavioural relaxation scale; pulse rate; Snoezelen Diary Card; Checklist of Challenging Behaviour; Behaviour Checklist Semi-structured interview with nurses Monitoring of medication and discharge rate	(see Chan et al., 2005) Perceived benefits: ↑ mood, interaction No effect on medication and discharge rate
14	Collier and Jakob, 2017 United Kingdom	Qualitative study: ethnographic design	4/5	1) *n* = 16 care homes, *n* = 32 staff interviewed 2) NA 3) dementia 4) residential	Appraise the evolving concept of MSEs from a user perspective, to study the aesthetic and functional qualities, to identify barriers to staff engagement with a sensory environment approach, and to identify design criteria	NA	Semi-structured interviews; observations of the MSE design	Perceived benefits in person with DM: ↑ interaction, relaxation; ↓ anxiety Perceived benefits in support person: ↑ relationship with participants
15	Collier et al., 2010 United Kingdom	RCT Control: gardening	2/5	1) *n* = 30 (MSE group: *M* = 80 yrs; *SD* 7.2; range 60–91) 2) 13F, 17 M 3) moderate to severe DM, and diagnosed sensory processing difficulties 4) residential	Explore the extent to which the sensory components of MSEs influence functional performance	1) NR 2) NR 3) 3x per week 4) NR 5) standardized in accordance with protocols describing presentation of equipment and structure and timing to participant's level of functioning; sensory processing preferences identified before intervention	Measure: Assessment of Motor and Processing Skills	Effect, but no difference from control condition: ↑ functional performance
16	Cuvo et al., 2001 United States	Non-randomized study: multiple-case design, alternating treatments Control: living room and outdoor activity	3/5	1) *n* = 4 (44, 48, 55, 65 yrs) 2) 2F, 2 M 3) profound ID, additional diagnoses include autism, seizure disorder, hyposcoliosis, tardive dyskinesia, pica and sleep disturbance and phenylketonuria 4) residential	Test the effect of a Snoezelen room on reducing stereotypic behaviour and increasing engagement	1) NR 2) individually 3) 5x per week 4) 20–45 min 5) introduction of stimuli followed by free movement around the room; no interaction unless requested by participant; if no engagement occurred after 2–4 min, the support person prompted the participant	Observation of operationalized stereotypic behaviour (body rocking, body swaying, picking, mouthing) and engagement (for example using materials, looking, touching)	Effect compared to living room, but outdoor activity more effective than snoezelen: ↓ stereotypic behaviour ↑ engagement
17	Fava and Strauss, 2010 Italy	Non-randomized study Control: living room and stimulus preference room	1/5	1) *n* = 27 (*M* = 37.8 yrs, range 30–48) 2) NR 3) profound ID, additional diagnosis includes autism (*n* = 9) 4) residential	Investigate whether the Snoezelen environment and the Stimulus Preference environment have differential effects on disruptive behaviour and pro-social behaviours and to study behavioural improvement in the naturalistic environment	1) to create a relaxing and safe atmosphere 2) individually 3) 3x per week 4) 25 min 5) non-directive approach; introduction of stimuli followed by free movement around the room; no interaction unless requested by the participants; if no engagement occurred after 4 min, the support person prompted the participant *The stimulus preference intervention used preferred stimuli and a directive approach*	Observation of disruptive behaviours (aggressive or stereotyped behaviours) and pro-social behaviours (active behaviours towards sensorial stimuli and social behaviours towards caregiver)	Effect compared to control in subgroup of individuals with autism: ↓ challenging behaviour Effect of stimulus preference room compared to snoezelen in subgroup with poor motor and linguistic abilities: ↑ pro-social behaviours
18	Goto et al., 2014 United States of America	Non-randomized study Control: indoor garden	0/5	1) *n* = 18 (*M* = 88) 2) 12F, 6 M 3) moderate to severe DM, and additional diagnoses including depression, Parkinson's disease, and coronary artery disease 4) residential	Determine the responses to snoezelen and a Japanese garden	1) NR 2) individually 3) 2x per week 4) 15 min 5) support person was able to choose the elements to be used according to the participant's needs	Behaviour Assessment Checklist; heart rate. Medical chart review	No effect on relaxation and interaction Effect in control group: ↑ relaxation, interaction
19	Hill et al., 2012 United Kingdom	Non-randomized study; multiple-case study, alternating treatments Control: living room	3/5	1) *n* = 2 (14,18 yrs) 2) 1F, 1 M 3) severe ID and additional diagnosis of autism. 4) school	Evaluate the effects of the MSE and the level of social contact provided on levels of stereotyped behaviours	1) NR 2) individually 3) 1x per week 4) 20–25 min 5) all equipment was switched on/off by support person; hand-held materials could be activated by participant or support person; high or low attention from the support person depending on study phase	Adaptive Behaviour Scale – School; Aberrant Behaviour Checklist; Diagnostic Assessment for the Severely Handicapped- II; Functional Assessment for Multiple Causality	Effect, irrespective of level of carer attention: ↓ challenging behaviour Effect under high carer attention in both snoezelen and living room: ↑ engagement
20	Hope, 1998 United Kingdom	Quantitative descriptive study No control	4/5	1) *n* = 29 (*M* = 76.7 yrs, range 54–91) 2) 21F, 8 M 3) dementia 4) residential, day centre	Evaluate how participants respond to the individual pieces of equipment in the MSE and evaluate the influence of the MSE on behaviour	1) NR 2) individually 3) NR 4) 30 min or more 5) protocol for internal session structure	Interact; pulse rate. Observational responses to equipment. Notes by staff on perceived effects	Perceived benefits: ↑ mood, interaction, relationship with support person
21	Hope et al., 2004 United Kingdom	Qualitative study; action research design	5/5	1) *n* = 15 staff 2) NA 3) dementia 4) National Health Service provision	Evaluate staff perspectives on their experiences of using the multisensory room, participants’ responses, implementation of workshops, and factors which helped or hindered the process	NA	Focus group and individual interviews	Perceived benefits in support people: ↑ awareness of participant's personhood, relationships with participants, positive changes in care delivery
22	Houghton et al., 1998 Australia	Non-randomized study No control	3/5	1) *n* = 17 (5–18 yrs) 2) 8F, 9 M 3) severe ID and multiple disabilities, additional diagnoses include autism, visual impairment, hearing impairment, and physical disability. 4) school	Study whether exposure to a MSE assists in achieving Foundation Outcome Statement Skills and assess whether this generalizes to other settings	1) NR 2) more than one 3) NR 4) 30–40 min 5) NR	Foundation Outcome Statement skills mapping instrument (for example skills related to awareness of self, social interactions, manipulation of objects and equipment, and communication)	Effect: ↑ Foundation Outcome Statement skills
23	Kaplan et al., 2006 United States of America	Non-randomized study: multiple-case study, alternating treatments Control: usual occupational therapy	1/5	1) *n* = 3 (31,47,52) 2) 1F, 2 M 3) moderate and profound ID and additional diagnosis of autism. 4) day centre	Investigate whether observed changes in engagement carried over to a post-session functional activity	1) to treat the proprioceptive and vestibular systems through directed auditory, tactile, and visual sensory input to effect arousal change 2) individually 3) 2x per week 4) 30 min 5) the participant and support person alternately adjusted the input	Observation of task engagement (number of prompts required to complete an individually specified task: colour bingo, sandwich making and eating, or playing catch) Observation of frequency of challenging behaviour (tantrums, crying and biting)	Effect in 2 out of 3 participants compared to control: ↑ engagement ↓ challenging behaviours No effect in 1 participant
24	Klages et al., 2011 Canada	RCT Control: volunteer visits	1/5	1) *n* = 19 (snoezelen: *M* = 84; *SD* 6.6) 2) 13F, 6 M 3) mild to severe DM 4) residential	Investigate the influence of snoezelen on balance and falls	1) stimulation and relaxation 2) individually 3) 2x per week 4) 30 min 5) preferences were taken into account; activities that stimulated tactile, visual, and proprioceptive sensations were encouraged; trusting relationship was developed; unstructured intervention	Functional Reach Test; Sharpened Romberg; Timed Up and Go test with and without cognitive dual task. Journal for balance-enhancing activities. Record of frequency of falls	No effect on standing balance and frequency of falls
25	Kwok et al., 2003 Hong Kong	Quantitative descriptive study No control	5/5	1) *n* = 96 (16–60 yrs) 2) male/female 3) mild to profound ID, additional diagnoses include epilepsy, hearing impairment, visual impairment, and psychiatric disorder 4) residential, day centre	Describe the use of a snoezelen (that is, multisensory) room and provide a subjective evaluation of the effectiveness of treatment	1) NR 2) NR 3) 1x per week 4) 60 min 5) NR	Rating form with 8 functional outcomes of snoezelen, which could be rated by three responses: no effect, mild effect, and marked effect	Perceived benefits: ↑ concentration, motivation for learning, self-confidence, mood, relaxation, relationship with support person; ↓ challenging behaviour
26	Lindsay et al., 2001 United Kingdom	Non-randomized study; crossover Control: relaxation therapy, hand massage/ aroma therapy, and activity sessions	2/5	1) *n* = 8 (23–62 yrs) 2) 6F, 2 M 3) profound ID 4) residential	Study the effects of four therapies on positive and negative forms of communication	1) NR 2) individually 3) NR 4) 20 min 5) NR	Communication rated on a five-point Likert scale consisting of 5 positive variables (friendly vocalization, soft touch, non-threatening gaze, laughter, and overall positive responsiveness) and 5 negative variables (screaming, self-injury, aggression to others, pulling away or leaving, and overall negative responsiveness)	Effect compared to hand massage/aroma therapy and activity sessions, but no difference from relaxation therapy: ↑ positive communication Slight decrease in negative communication in snoezelen and relaxation therapy
27	Lindsay et al., 1997 United Kingdom	Non-randomized study; crossover Control: relaxation therapy, hand massage/ aroma therapy, and activity sessions	2/5	1) *n* = 8 (*M* = 38.6 yrs; 23–62) 2) 6F, 2 M 3) profound ID 4) residential	Compare four therapies on the extent to which they foster relaxation and help participants to concentrate on adaptive tasks	1) NR 2) individually 3) NR 4) 20 min 5) NR	Assessment of concentration based on the number of movements made to engage in a 5 min task Rating of enjoyment/ relaxation on a scale from 0 (no response) to 4 (very responsive to treatment/ enjoying treatment a great deal)	Effect compared to hand massage/aroma therapy and activity sessions, but no difference from relaxation therapy: ↑ concentration No effect on enjoyment/ relaxation, though both snoezelen and relaxation therapy were perceived as the most enjoyable therapies for participants
28	Lo Buono, Torrisi, Leonardi, Pidalà, and Corallo, 2022 Italy	Non-randomized study; single-case study No control	Failed screening criteria	1) *n* = 1 (7 yrs) 2) 1 M 3) severe ID, spastic quadriplegia 4) clinical rehabilitation centre	Examine the effect of snoezelen on rehabilitation	1) relaxing and stimulation 2) individually 3) 2x per week 4) 60 min 5) activities were provided in a direct manner, as opposed to non-directive.	Neurological examination; pediatric Functional Independence Measure; Vineland Adaptive Behaviour Scale	Effect: ↓ self-harm and motor stereotypies ↑ sustained attention ↑ involvement in activities
29	Lorusso et al., 2022 United States of America	Non-randomized study; single-case study, multiple baseline Control: usual care	2/5	1) *n* = 4 (62, 69, 72, 80 yrs) 2) 1F, 3 M 3) dementia, additional diagnoses include mental health disorders, and Huntington's disease. 4) military long-term care facility	Evaluate the impact of MSEs on behaviour within the bathing environment	1) achieve sensory balance 2) individually 3) 2x per week 4) NR 5) preference assessment of music and aroma's.	Observation of positive and challenging behaviours	Effect compared to control: ↑ engagement ↑ mood ↓ challenging behaviours
30	Lorusso et al., 2020 United States of America	Qualitative study	5/5	1) *n* = 32 2) 28F, 4 M 3) NA (staff): snoezelen used in veterans with dementia 4) 12 different sites	Explore staff perceptions of the effectiveness of MSEs	NA	21 individual interviews and one group interview with 11 participants	Perceived benefits: ↑ positive distraction, engagement, relaxation ↓ challenging behaviour Perceived benefits for staff: ↑ relaxation Perceived negative effects: increased negative emotions (for example, scared or upset)
31	Lotan et al., 2009 Israel	Non-randomized study; multiple-case study Control: washout period with usual care	Failed screening criteria	1) *n* = 10 (*M* = 45 yrs; *SD* 16.45; range 28–74) 2) 4F, 6 M 3) moderate to severe ID, additional diagnoses include Fragile-X syndrome, Angelman syndrome, and hearing impairment. 4) residential	Investigate the efficacy of the MSE as an appropriate, accepted therapeutic tool to reduce challenging behaviour	1) provide relaxing atmosphere 2) individually 3) 2x per week 4) 30 min 5) enabling approach; preference assessment during the first session; the participant received the same individually selected environment on repeated visits (ranging from lying on the waterbed for half an hour with no contact, to a full body massage)	Challenging behaviour was recorded on an individually adopted chart	Effect compared to control: ↓ challenging behaviours
32	Machado and Castro, 2022 Brazil	Non-randomized study Control: usual care	1/5	1) *n* = 20 (*M* = 83 yrs) 2) 17F, 3 M 3) moderate or severe DM, additional diagnoses include depression, Parkinson's disease, and stroke 4) nursing home	Investigate the effect of a multisensory programme on behavioural, mood, and biomedical parameters	1) provide excitatory or relaxing stimuli 2) individually 3) 2x per week 4) 30 min 5) non-directive approach, preference assessment in first session, possibility during sessions to transfer between various sensory spaces (garden, room, corridor),	Cornell Scale for Depression in Dementia; Neuropsychiatric Inventory; Checklist on behaviour, mood, interaction with the environment and caregiver; physiological measures	Effect, but no difference from control: ↓ neuropsychiatric symptoms Some improvement in cognition in the intervention group, and improved mood in both groups. Perceived benefits: ↑ social interaction and engagement
33	Mahdavi et al., 2015 Iran	RCT Control: usual care	1/5	1) *n* = 40 (snoezelen: *M* = 66 yrs) 2) NR 3) dementia 4) treatment clinic	Discuss effectiveness of multisensory stimulations on restoration of patients exposed vascular dementia	1) NR 2) NR 3) NR 4) 60 min 5) NR	Measures: Mini-Mental State Examination	Effect compared to control: ↑ cognitive status
34	Martin et al., 1998 United Kingdom	RCT; double crossover Control: activity modelled on what happened in the MSE, but in a room furnished with a table and chair	3/5	1) *n* = 27 (*M* = 38 yrs; range 22–61) 2) 9F, 18 M 3) severe to profound ID 4) day centre	Evaluate the effects of MSEs on challenging behaviour	1) NR 2) group (of 3, 4 or 5 participants) 3) 2x per week 4) 60 min 5) the enabler gave each participant 6 min of attention. When the enabler had given attention to everyone in the subgroup, the sequence was repeated.	Functional Performance Record, Problem Behaviour Inventory Observation of task-related and challenging behaviours using four analogue conditions (alone, contingent attention, non-contingent attention, demand)	No effect on challenging behaviour between the MSE and control condition
35	Maseda et al., 2018 Spain	RCT Control: music sessions	1/5	1) *n* = 21 (*M* = 88.9 yrs; *SD* 6.69; range 77–102) 2) 15F, 6 M 3) severe or very severe DM 4) residential	Assess whether multisensory stimulation in a Snoezelen room is more effective than individualized music sessions in terms of mood, behaviour, and biomedical parameters	1) NR 2) individually 3) 2x per week 4) 30 min 5) non-directive enabling approach; support persons encourages patients to engage with sensory stimuli of their choice; non-sequential and unpatterned stimuli; not relying on short-term memory; internal session structure; sensorial preferences and interests were collected previously	Interact Scale; Interact Short; heart rate and saturation	Effect, but no difference from control: ↑ interaction On item level, snoezelen was more effective than control on visual follow-up of stimuli. And music more effective than snoezelen on relaxation and happiness.
36	Maseda et al., 2014 Spain	RCT Control: activity sessions, usual care	1/5	1) *n* = 30 (*M* = 87.3 yrs; *SD* 5.3) 2) 27F, 3 M 3) mild to severe DM 4) residential	Evaluate effectiveness of the MSE in terms of behaviour, mood, and cognitive and functional impairment in basic activities of daily living	1) NR 2) individually 3) 2x per week 4) 30 min 5) non-directive approach; efforts to stimulate all senses except taste; unpatterned and nonsequential stimuli; no intellectual/physical demands; internal session structure; sensorial preferences and interests were collected previously	Cohen-Mansfield Agitation Inventory; Neuropsychiatric Inventory – Nursing Home; Cornell Scale for Depression in Dementia; Mini Mental State Examination; Global Deterioration Scale; Barthel Index	Effect, but no difference from activity sessions: ↓ agitation, neuropsychiatric symptoms On item level, snoezelen increased physically nonaggressive behaviour compared to activity sessions; no difference from usual care No effect on cognitive status, functional performance, dementia severity and mood.
37	Maseda et al., 2014 Spain	RCT Control: activity sessions	1/5	1) *n* = 20 (*M* = 87.5 yrs; *SD* 5.7; range 77–96) 2) 19F, 1 M 3) mild to severe DM 4) residential	Assess whether multisensory stimulation in a Snoezelen room is more effective than one-to-one activity sessions in terms of mood, behaviour, and biomedical parameters	1) NR 2) individually 3) 2x per week 4) 30 min 5) non-directive approach; unpatterned stimuli; few intellectual/physical demands; internal session structure; sensorial preferences and interests were collected previously	Interact Scale; Interact Short; heart rate and saturation	Effect, but no difference from control: ↑ mood, relaxation, interaction
38	McKee et al., 2007 Canada	Non-randomized study; multiple-case study, alternating treatment Control: usual care	1/5	1) *n* = 3 (28,31,32 yrs) 2) 3 M 3) moderate ID and additional diagnosis of autism. 4) residential	Examine the effect of snoezelen on aggressive and destructive behaviour	1) NR 2) individually 3) 5x per week 4) 45 min 5) the facilitator followed the participant's lead and engaged with him in whatever was of interest. Otherwise, the support person sat quietly and offered back rubs.	Observation and recording of occurrence of disruptive and prosocial target behaviours	No effect in 2 out of 3 participants on challenging behaviour Negative effect in 1 out of 3 participants: ↑ challenging behaviour Slight increase in prosocial behaviours
39	Milev et al., 2008 Canada	RCT Control: usual care	0/5	1) *n* = 21 (*M* = 84.4 yrs; *SD* 6.23; range 73–94) 2) 15F, 3 M 3) severe DM 4) residential	Examine whether multisensory stimulation sessions had a beneficial effect on behaviour as opposed to care as usual	1) NR 2) individually 3) 1x per week or 3x per week (two subgroups) 4) 30 min 5) if participants became unhappy while interacting with something in the room, they were promptly shown something else	Daily Observation Scale (asleep in bed, asleep in chair, awake and calm, agitated, in Life Enrichment Programme, engaged with others, sitting alone, alone in room); Clinical Global Impression Improvement	Effect compared to control: ↑ mood, interaction Increase in sessions shows a trend for better outcomes
40	Minner et al., 2004 United States of America	Quantitative descriptive study; quality improvement project No control	Failed screening criteria	1) *n* = 19 2) NR 3) dementia 4) residential	Evaluate whether use of snoezelen therapy could reduce the number of behavioural symptoms	1) NR 2) individually (1 case description) 3) 3x per week (1 case description) 4) 30 min (1 case description) 5) NR	Comfort/Discomfort Scale with added positive behaviours (for example, content facial expressions, positive interactions with people or objects)	Effect: ↑ mood, interaction
41	Mitchell et al., 2015 United States of America	Non-randomized study No control	2/5	1) *n* = 13 (*M* = 79.5 yrs; *SD* 8.6; range 61–89) 2) 9F, 4 M 3) dementia 4) residential	Examine the usefulness of the multisensory room intervention on agitation	1) NR 2) NR 3) NR 4) 15 to 30 min 5) support person exposes participant to non-pharmacological therapeutic interventions in the multisensory room	Measures: Pittsburgh Agitation Scale	Effect: ↓ agitation
42	Moir, 2010 Australia	Non-randomized study: multiple-case study Control: intervention in the natural environment for one participant	3/5	1) *n* = 3 (1 yr, 10mo; 2 yrs, 6 mo; 4 yrs, 9 mo) 2) 1F, 2 M 3) severe to profound multiple disabilities, additional diagnoses include epilepsy, visual impairment, and motor disabilities. 4) early childhood development programme	Evaluate the effects of learning switching skills using a multisensory environment and the child's natural environment	1) NR 2) individually 3) NR 4) 15–33 min 5) use of teaching protocols; using prompts where necessary to assist learning	Switching and Associated Behavioural Responses Schedule Follow-up informal qualitative interviews	Effect compared to control participant: ↑ using switch skills Qualitative findings indicate increased adaptive behaviours in all 3 participants
43	Nasser et al., 2004 Israel	Qualitative study: social work project	Failed screening criteria	1) *n* = 47 families 2) NA 3) severe to profound ID, additional diagnoses include Down syndrome, epilepsy, blindness, and motor disabilities. 4) residential	Evaluate the use of snoezelen in working with the whole family	1) to facilitate family encounters 2) group (family) 3) NR 4) 35–70 min 5) first part (20–40 min) was free activity, second part more structured (15–30 min). The occupational therapist guided activities, while the social worker participated, answered questions, and addressed issues that arose.	Data was gathered through conversations with parents, siblings, and discussions amongst professionals	Perceived benefits: ↑ family occupations, relationships within the family
44	Novakovic et al., 2019 Serbia	RCT Control: usual care	2/5	1) *n* = 40 (15–35 yrs) 2) ‘both sexes’ 3) mild to profound ID and additional diagnosis of autism. 4) day centre	Determine the effects of snoezelen on the severity of autism spectrum disorder and specifically stereotyped/ repetitive behaviours	1) to have participants gradually take over the activities and slowly accept them, which stimulated the senses 2) group (of 3 participants) 3) 3x per week 4) 30 min 5) non-directive approach; after the stimuli were introduced, the participants were encouraged to move around; participants were free to choose equipment	Childhood Autism Rating Scale	Effect compared to control: ↓ severity of autism symptoms and repetitive and stereotyped behaviours
45	Prince et al., 2022 United States of America	Non-randomized study: retrospective medical record analysis Control: usual care	0/5	1) *n* = 24 (over 65 yrs) 2) 19F, 5 M 3) dementia, additional diagnoses include motor disability 4) memory-care assisted living facility	Examine the outcomes of an accessible multisensory room on episodes of behavioural and psychological symptoms of dementia	1) NR 2) NR 3) participants could enter or exit at any time 4) NR 5) the multisensory room as an area within the living unit (open floorplan).	Reporting of behavioural and psychological symptoms of dementia in the residents’ medical record	Effect compared to control: ↓ behavioural and psychological symptoms of dementia
46	Sachs and Nasser, 2009 Israel	Qualitative study: phenomeno-logical approach	5/5	1) *n* = 10 families (children with ID 4–17 yrs) 2) NA 3) severe to profound ID and additional diagnoses including motor disabilities. 4) residential	Understand the meaning of family occupations in the snoezelen environment for parents and other family members	1) NR 2) group (family) 3) NR 4) NR 5) NR	Semi-structured, in-depth interviews and participant observations	Perceived benefits: ↑ quality of family encounters due to experiencing relaxation and intimacy. Snoezelen fostered the experience of being together as a family.
47	Safavi et al., 2013 Iran	RCT Control: usual care	2/5	1) *n* = 52 (experimental group: *M* = 68.27 yrs) 2) 52F 3) mild to moderate DM 4) elderly care centre	Determine the effect of multi-sensory stimulation on cognitive status	1) NR 2) NR 3) NR 4) 45 to 60 min 5) non-directive enabling approach; attempts to stimulate all of the senses	Mini Mental State Examination; Brief cognitive status exam	Effect compared to control: ↑ cognitive status
48	Sánchez et al., 2016 Spain	RCT Control: activity sessions, usual care	1/5	1) *n* = 32 (*M* = 85.4 yrs; *SD* 8.64; range 68–102) 2) 25F, 7 M 3) severe or very severe DM 4) residential	Compare the effect of multisensory stimulation in a Snoezelen room and one-to-one activity sessions on behaviour, mood, cognitive status, and dementia severity	1) NR 2) individually 3) 2x per week 4) 30 min 5) non-directive approach; stimulate all of the senses except taste; unpatterned stimuli; few intellectual or physical demands; preferences and interests were collected previously	Cohen-Mansfield Agitation Inventory; Neuropsychiatric Inventory; Cornell Scale for Depression in Dementia; Severe Mini-Mental State Examination; Bedford Alzheimer Nursing Severity Scale	Effect compared to usual care and activity sessions: ↓ dementia severity Effect compared to activity sessions, but no difference from usual care; ↓ neuropsychiatric symptoms Effect compared to usual care, but no difference from activity sessions: ↓ agitation No effect on cognitive status and mood
49	Sánchez et al., 2016 Spain	RCT Control: music sessions	1/5	1) *n* = 22 (*M* = 88.41 yrs; *SD* 6.93; range 77–102) 2) 15F, 7 M 3) severe or very severe DM 4) residential	Compare the effects of multisensory stimulation in a Snoezelen room and individualized music sessions on agitation, emotional and cognitive status, and dementia severity	1) NR 2) individually 3) 2x per week 4) 30 min 5) non-directive enabling approach; encouraging participants to engage with sensory stimuli of their choice; non-sequential and unpatterned stimuli; not relying on short-term memory; internal session structure; sensorial preferences and interests were collected previously	Cohen-Mansfield Agitation Inventory; Cornell Scale for Depression in Dementia; Rating Anxiety in Dementia; Severe Mini-Mental State Examination; Bedford Alzheimer Nursing Severity Scale	Effect compared to control: ↓ anxiety, dementia severity Effect, but no difference from control: ↓ agitation No effect on cognitive status or mood
50	Shapiro et al., 1997 Israel	RCT; crossover Control: playroom	2/5	1) *n* = 20 (*M* = 7.5 yrs, range 5–10) 2) 5F, 15 M 3) moderate or severe ID 4) sample institution	Determine the efficacy of snoezelen on maladaptive behaviours	1) NR 2) individually 3) NR 4) 20 min 5) sessions are child centred and consist of a flexible sequence	The Behaviour Checklist; heart rate	Effect compared to control: ↓ maladaptive behaviours; ↑ adaptive behaviours Changes in heart rate (both directions occurred)
51	Singh et al., 2004 United States of America	Non-randomized study; counterbalanced design Control: ADL skills training, vocational skills training	1/5	1) *n* = 45 (22–57 yrs) 2) 14F, 31 M 3) severe or profound ID and additional psychiatric disorder (for example, schizophrenia or obsessive compulsive disorder) 4) residential	Study the effect of snoezelen, ADL skills training, and vocational skills training on aggression and self-injury	1) NR 2) group (of 15 participants) 3) 5x per week 4) 60 min 5) NR	Observation of occurrence and non-occurrence of aggressive acts and self-injurious behaviours	Effect compared to both controls: ↓ aggression Effect compared to ADL skills training, but no difference from vocational training: ↓ self-injury
52	Slevin and McClelland, 1999 United Kingdom	Non-randomized study; single-case study No control	2/5	1) *n* = 1 (22 yrs) 2) M 3) severe ID and additional diagnosis of autism 4) residential	Investigate whether a multisensory environment induced relaxation	1) NR 2) individually 3) 7x per week 4) 20 min 5) no prompting; interactions were avoided	Behavioural Relaxation rating Scale; pulse rate Nursing notes for recorded incidents of challenging behaviour	Effect: ↑ relaxation No effect on frequency of challenging behaviour, though behaviours were less severe
53	Spaull et al., 1998 United Kingdom	Non-randomized study; multiple-case study, alternating treatment Control: usual care	0/5	1) *n* = 4 (77,79,82,84 yrs) 2) 4 M 3) severe DM, and additional diagnoses including psychiatric disorders 4) residential	Investigate the effect of sensory stimulation on behaviour, adaptive functioning, and general wellbeing	1) NR 2) individually 3) NR 4) NR 5) freeform intervention dictated by the participant's response to the stimuli	Measures: Modified Behaviour Rating Scale; Short form Adaptive Behaviour Scale; Dementia Care Mapping	Effect compared to control: ↑ interaction, adaptive skills No effect on wellbeing
54	Staal et al., 2007 United States of America	RCT Control: recreational activity therapy	1/5	1) *n* = 24 (intervention group: *M* = 80.33 yrs; *SD* 1.59) 2) 16F, 8 M 3) moderate to severe DM 4) residential	Assess whether a combined treatment reduces agitation and apathy and improves activities of daily living	1) NR 2) individually 3) NR 4) 15, then 20–25, to 30 min 5) graded introduction to multi-sensory environment; 2 to 3 sessions to assess sensory preferences; expand duration of sessions using fixed time intervals	Global Deterioration Scale; Pittsburgh Agitation Scale; Multi-level Assessment Instrument, one subscale (Physical Health); Scale for the Assessment of Negative Symptoms in Alzheimer's Disease; Katz Index of Activities of Daily Living; Refined Activities of Daily Living Assessment Scale; Mini Mental Status Exam	Effect compared to control: ↑ functional performance of activities of daily living ↓ agitation, dementia severity
55	Stephenson and Carter, 2011a Australia	Qualitative study	5/5	1) *n* = 5 teachers 2) NR 3) NA (teachers); snoezelen used in severe ID, and additional diagnoses including autism and motor disabilities 4) school	Find out perceived benefits, encountered problems, use of the MSE, programming for sessions, observed outcomes, and how teachers learned to use the MSE with students with severe ID	NA	Interviews with 5 teachers and observations in the MSE	Perceived benefits: ↑ relaxation, mood, concentration, motivation to learn, learning skills, building relationships, mood, interaction with the environment (for example visual tracking)
56	Stephenson and Carter, 2011b Australia	Qualitative study	4/5	1) *n* = 4 staff 2) NR 3) NA (staff); snoezelen used in severe ID, and additional diagnoses including motor disabilities 4) 2 schools	Explore the background to the installations of MSEs, perceptions, and beliefs about the effects of use of MSEs with students with severe ID	NA	Interviews with 4 staff from 2 schools	Perceived benefits: ↑ learning skills (for example cognitive, physical, interaction), relaxation, concentration, motivation, choice making, mood, interaction, building relationships
57	Toro, 2019 Italy	Non-randomized study; crossover Control: watching television, usual care	3/5	1) *n* = 35 (25–72 yrs) 2) 7F, 29 M 3) moderate ID, and additional diagnoses including epilepsy, Down syndrome, psychiatric disorders and brain injury. 4) residential	Explore whether multisensory stimulation has a subsequent improvement on memory and standing balance	1) relaxation sessions 2) individually 3) 1x per week 4) 20 min 5) support person ensured that the participants rested on the waterbed, but remained neutral and non-directive throughout	Digit span test; Romberg and Sharpened Romberg test	Effect compared to both controls: ↑ memory, standing balance
58	Tunson and Chandler, 2010 United States of America	Non-randomized study; multiple-case study, alternating treatment Control: usual classroom	2/5	1) *n* = 3 (3,7,10 yrs) 2) 1F, 2 M 3) severe multiple disabilities, and additional diagnoses including seizure disorder, cerebral palsy, and scoliosis. 4) school	Examine responsiveness within and outside a MSE	1) NR 2) group (in classroom) 3) 3x per week 4) 30 min 5) NR	Observation of alertness and responsiveness at 10 min intervals (asleep, awake/agitated, awake/inactive, self-directed, visually attentive, active reaching)	No effect in 2 out of 3 people on alertness and responsiveness Effect in 1 out of 3 people: ↑ response to the environment instead of self-directed behaviour
59	Van der Putten et al., 2011 The Netherlands	Non-randomized study No control	2/5	1) *n* = 23 children (*M* = 11.6 yrs, *SD* 3.2) *n* = 3 teachers (27,45,52 yrs) 2) children: 11F, 12 M 3) profound intellectual and multiple disabilities, including motor disabilities 4) school	Investigate whether and, if so, to what extent the teacher's knowledge of the child's sensory and motor abilities and contextual preferences increases by using the MSE	1) NR 2) individually 3) 2x per week 4) 20 min 5) teacher carried out individual activities with the child; teachers could choose materials to stimulate the senses	Inventory for tuning activities and situations to the abilities and preferences of children with profound intellectual and multiple disabilities	Effect: ↑ teachers knowledge of sensory abilities and contextual preferences of children
60	Van Diepen et al., 2002 United Kingdom	RCT Control: reminiscence therapy	1/5	1) *n* = 10 2) NR 3) moderate to severe DM 4) day centre	Evaluate the feasibility of using a detailed approach to behavioural and physiological assessments before, during, and after snoezelen sessions and secondary to identify effects	1) create a relaxing but stimulating atmosphere 2) individually 3) 2x per week 4) 40 min 5) support person facilitates rather than directs the participant to explore the environment	Mini Mental State Examination; Clinical Dementia Rating; Cohen-Mansfield Agitation Inventory; Agitation Behaviour Mapping Instrument; Interact scale; heart rate	Effect, but no difference from control: ↑ mood ↓ agitation Participant's heart rate both increased and decreased during sessions
61	Vlaskamp et al., 2003 The Netherlands	Non-randomized study: multiple-case study Control: living room	3/5	1) *n* = 19 (*M* = 28 yrs, range 18–41) 2) 11F, 8 M 3) profound intellectual and multiple disabilities, including motor disabilities 4) facilities	Investigate whether the use of MSEs resulted in increased alertness or interaction amongst people with profound multiple disabilities	1) being active (to increase the level of interaction and alertness) 2) group (2–7 participants) 3) NR 4) 30 min 5) Staff members chose materials to increase level of alertness and interaction. Most material was presented continuously. Stimuli from staff were offered non-continuously.	Observation of interaction and alertness at 30 second intervals (A) asleep, inactive, not alert; B) awake, inactive, not alert; C) active, self-directed; D) sensory active, directed at environment; E) sensory and motor active, directed at environment	No effect on level of activity In both settings, non-continuous stimuli were usually associated with alertness or interaction
62	Ward-Smith et al., 2009 United States of America	Non-randomized study: retrospective medical record audit Control: usual care	1/5	1) *n* = 14 (*M* = 81.3 yrs; *SD* 7.8; range 67–92) 2) 12F, 2 M 3) dementia 4) residential	Compare the incidences of problematic behaviour in participants who were and were not exposed to an MSE	1) to provide relaxation and enhance alertness 2) individually 3) NR 4) 15 to 20 min 5) treatment plan tailored to each participant	Psychotic Behaviour Assessment Record	Effect compared to control: ↓ in incidences of disruptive behaviour, but not the behaviours present

Note: grey lines are studies involving people with dementia; white lines: people with intellectual disability; *n* = number of participants; ID = intellectual disability; DM = dementia; *M* = mean; SD = standard deviation; yrs = years; *F* = female; *M* = male; MSE(s) = multisensory environment(s); ADL = activities of daily living; NA = not applicable; NR = not reported; RCT = randomized controlled trial.

### 4.2 Study characteristics

In total, 53 of the studies (85.5%) used a quantitative design, eight studies (12.9%) a qualitative design, and one study (1.6%) used mixed methods. Five studies (8.1%) scored a maximum of 5 out of 5 on the quality appraisal (scored as ‘yes’); the average score was 1.8 out of 5. Many quantitative studies (75.8%) left out details about one or more criteria; those criteria could not be appraised positively or negatively (scored as ‘can't tell’).

In 23 studies, at least one control group was used where participants did not receive snoezelen (intellectual disability *n* = 5, 16.7%; dementia *n* = 18, 56.3%). In 21 studies, all participants received snoezelen and at least one control condition, such as usual care, alternating treatment, or crossover designs (intellectual disability *n* = 14, 46.7%; dementia *n* = 7, 21.9%). Ten quantitative studies were non-controlled (intellectual disability *n* = 6, 20.0%; dementia *n* = 4, 12.5%). Almost all studies evaluated the effect of snoezelen during or immediately after the application (intellectual disability *n* = 23, 76.7%; dementia *n* = 22, 68.8%). Long-term effects were evaluated in eight studies, ranging from one to five months follow-up (intellectual disability *n* = 2, 6.7%; dementia *n* = 6, 18.8%).

### 4.3 Participant characteristics

Snoezelen was mainly used in residential (*n* = 43, 69.4%) and day-care (*n* = 11, 17.7%) settings. In addition, school settings (*n* = 8, 12.9%) were reported in studies involving people with intellectual disability.

The number of participants ranged from 1 to 136, with an average of 26 participants. Studies included participants between the ages of 2 to 74 years (intellectual disability) and 54 to 102 years (dementia). Two articles involving people with intellectual disabilities used an age group of “over 71″ without specifying the exact age. Studies included participants whose level of intellectual disability ranged from mild to profound and whose level of dementia severity ranged from mild to very severe. Reports on participant characteristics also included additional impairments (intellectual disability *n* = 22, 73.3%; dementia *n* = 8, 25.0%), such as sensory or motor impairment. Additional impairments were not reported or were unclear in 32 studies (intellectual disability *n* = 8, 26.7%; dementia *n* = 24, 75.0%).

### 4.4 Purpose of snoezelen

In 15 studies (24.2%), the reason for using snoezelen was clearly described: five studies used snoezelen for relaxation (intellectual disability *n* = 3, 10.0%; dementia *n* = 2, 6.3%), six for relaxation and activation (intellectual disability *n* = 1, 3.3%; dementia *n* = 5, 15.6%), and one for activation (intellectual disability *n* = 1, 3.3%). Two studies used snoezelen to affect arousal levels in an unreported direction (intellectual disability *n* = 2, 6.7%). Lastly, the intervention purpose in one study was to facilitate family encounters (intellectual disability *n* = 1, 3.3%).

### 4.5 Application of snoezelen

In general, 10 application characteristics of snoezelen were identified in the studies; see Table 3. For a detailed reference to specific studies, we refer to supplementary material, S-Table II.

**Table 3:** Number of studies reporting on characteristics of application.

Element	Characteristic of application	ID (*n* = 30) *n* (%)	DM (*n* = 32) *n* (%)	Total *n* (% of 62)
Application of multisensory stimuli	Senses addressed Materials and equipment used Frequency of session(s) Duration of session(s) Strategies in applying stimuli	16 (53.3) 28 (93.3) 20 (66.7) 27 (90.0) 19 (63.3)	12 (37.5) 26 (81.3) 19 (59.4) 23 (71.9) 19 (59.4)	28 (45.2) 54 (87.1) 39 (62.9) 50 (80.6) 38 (61.3)
Multisensory environment	Physical aspects of the MSE Social context in the MSE	17 (56.7) 25 (83.3)	4 (12.5) 22 (68.8)	21 (33.9) 47 (75.8)
Support during snoezelen	Presence of support person Training of support person Role of support person	30 (100) 7 (23.3) 21 (70.0)	32 (100) 14 (43.8) 11 (34.4)	62 (100.0) 21 (33.9) 32 (51.6)

Note: *n* = number of studies; ID = intellectual disability; DM = dementia; MSE = multisensory environment.

#### 4.5.1 Application of multisensory stimuli

The senses addressed were mentioned in nearly half of all the studies (*n* = 28, 45.2%). The visual (*n* = 28, 45.2%) and tactile (*n* = 28, 45.2%) senses were most commonly addressed for both intellectual disabilities and dementia, followed by auditory (*n* = 27, 43.5%), olfactory (*n* = 23, 37.1%), and gustatory (*n* = 2, 3.2%). Proprioception (*n* = 5, 8.1%) or vestibular stimuli (*n* = 6, 9.7%) were reported in a small number of studies.

Most studies (*n* = 54, 87.1%) described the specific materials and equipment that were used during snoezelen. The most frequently named material was a music player (*n* = 48, 77.4%), followed by a bubble tube (*n* = 41, 66.1%), projection equipment (*n* = 41, 66.1%), aromatics (*n* = 39, 62.9%), fibre optic material (*n* = 36, 58.1%), tactile material (*n* = 26, 41.9%), mirrors (*n* = 23, 37.1%), and vibrating material (*n* = 17, 27.4%). The use of water/music beds and bean bags was particularly mentioned in studies involving people with intellectual disabilities (*n* = 19, 63.3%; dementia *n* = 10, 31.3%).

The frequency of snoezelen was described in 39 studies (62.9%) and ranged from once to seven times a week. The most common frequency was twice a week (*n* = 19, 30.6%). The duration of sessions was reported in 50 studies (80.6%) and ranged from 5 to 120 min, with a mode of 30 min (*n* = 22, 35.5%). Studies involving people with intellectual disabilities more frequently reported (*n* = 11, 36.7%) a duration longer than 30 min than studies involving people with dementia (*n* = 6, 18.8%).

The structure of the session (for example, introducing, maintaining, and winding down a session) was reported in 15 studies (24.2%); however, except for three studies that described in detail how they introduced stimuli at the beginning of each session, details were lacking. Strategies regarding the application of stimuli were reported in 38 studies (61.3%). Apart from naming the strategy, few details were provided. Strategies included, for example, the use of a preference assessment (*n* = 12, 19.4%), the use of a non-directive approach (*n* = 21, 33.9%), and not placing physical or intellectual demands on participants (*n* = 8, 12.9%). Eight studies (12.9%) concerning people with dementia specifically mentioned the provision of stimuli in a non-structured and non-sequential manner, although the application was not explained.

#### 4.5.2 Multisensory environment

Four studies (7.0%) reported the use of a temporarily adapted space; namely, an adapted hall, classroom, or staff office. One third of the studies reported physical aspects of the multisensory environment (intellectual disability *n* = 17, 56.7%; dementia *n* = 4, 12.5%). Of these, 15 studies (24.2%) reported the size of the room, ranging from 6m^2^ to 68m^2^, with an average of 27m^2^. The most frequently reported physical aspects were a white room colour (*n* = 10, 16.1%), padded walls or floors (*n* = 7, 11.3%), and blocked daylight (*n* = 7, 11.3%). In most studies, snoezelen was an individual activity (*n* = 36, 58.1%). Snoezelen in a group (with more than one participant) was mainly mentioned in studies involving people with intellectual disabilities (*n* = 11, 36.7%; dementia *n* = 2, 6.3%). In six studies, the group size ranged from two to seven people, and one study used snoezelen in a group of 15 people. In six of these 13 studies, the number of people in the group was unclear (for example, classroom, family).

#### 4.5.3 Support person

All studies reported the presence of a professional during snoezelen, such as a therapist (for example, occupational therapist) (*n* = 20, 32.3%) or a carer/nurse (*n* = 17, 27.4%). In two studies (3.2%), family members participated in snoezelen together with a professional. One study involving people with dementia reported that professionals were mostly present, but intervention could also be provided without a professional present. Twenty-one studies (33.9%) reported that the support person was trained in the use of snoezelen. Most of these studies did not provide details on the training (*n* = 12, 19.4%); if details were provided, these included, for example, a demonstration of the room.

Information on the role of the support person during snoezelen was missing in half of the studies. Studies that gave more information reported that the role of the support person was more often active (for example, providing massages, engaging participants) (*n* = 28, 45.2%) than passive (for example, supervising, avoiding interaction) (*n* = 11, 17.7%). Both passive and active characteristics could also be addressed in the same study, for example, by first introducing stimuli and then allowing participants to explore freely with minimum interaction.

### 4.6 Effects of snoezelen

The effects of snoezelen reported in both quantitative and qualitative studies are summarized in Table 4. In total, 52 studies (83.9%) reported positive effects of snoezelen on one or more outcome measures, 18 studies (29.0%) reported no effects, and four studies reported negative effects (6.5%). Most effects were reported in the mental and physical health dimension (intellectual disability *n* = 18, 60.0%; dementia *n* = 21, 65.6%), such as improved mood, and the participation dimension (intellectual disability *n* = 10, 33.3%; dementia *n* = 13, 40.6%), such as increased engagement and interaction with the social and physical environment. Effects occurred during or immediately after snoezelen. Of the eight studies that evaluated longer-term effects, one study reported lasting positive effects on mood and engagement in people with dementia 12 weeks after intervention (Milev et al., 2008). Seven studies reported no long-term effects of snoezelen on various outcome measures mainly involving mental health and participation. Two studies suggested a need to use snoezelen continuously because of the disappearance of positive effects in the follow-up period (Lotan et al., 2009; Sánchez et al., 2016).

**Table 4:** Reported effects of snoezelen per target group classified in accordance with the human functioning dimensions the AAIDD model (the reference numbers correspond to Table 2).

		Effect of snoezelen		No effect of snoezelen	
AAIDD Dimension	Outcome	Intellectual disability	Dementia	Intellectual disability	Dementia
**Intellectual functioning**	Cognitive status		*n* = 2		*n* = 4
			More effective than usual care (33, 47)		On a par with usual care (36, 48), activity sessions (6, 36, 48), or music sessions (49)
	Concentration	*n* = 5			
		More effective than hand massage/aromatherapy or active therapy (27). On a par with relaxation therapy (27).			
	Memory	*n* = 1			
		More effective than television condition (57)			
**Adaptive behaviour**	Adaptive skills	*n* = 2	*n* = 1	*n* = 1	
		More effective than playroom condition (50)	More effective than usual care (53)	On a par with activity sessions (12)	
	Communication	*n* = 1			
		More effective than hand massage/aromatherapy or active therapy (26). On a par with relaxation therapy (26).			
	Functional performance		*n* = 2		*n* = 1
			More effective than indoor gardening (15) or activity sessions (54)		On a par with usual care or activity sessions (36)
	Foundation Outcome Statement Skills	*n* = 1			
		NA			
	Using a switch	*n* = 1			
		More effective than usual care (42)			
**Health** **(mental)**	Agitation		*n* = 8		*n* = 1
			More effective than usual care (36, 48). On a par with reminiscence (3, 4, 60), activity sessions (36, 48, 54), or music sessions (49).		On a par with an exercise programme (9)
	Anxiety		*n* = 2		
			More effective than music sessions (49)		
	Autism symptoms	*n* = 1			
		More effective than usual care (44)			
	Challenging behaviour	*n* = 11	*n* = 3	*n* = 4	
		More effective than usual care (16, 17, 28), living room with varied carer attention (19), a playroom (50), skills training (51), regular occupational therapy (23), or a structured stimulus preference room (17). Less effective than an outdoor activity (16).	More effective than usual care (29, 62)	On a par with usual care (11, 38*), massage therapy (11), or activity sessions (12, 34)	
	Dementia severity		*n* = 4		*n* = 1
			More effective than usual care (45, 48), activity sessions (48, 54), or music sessions (49)		On a par with usual care or activity sessions (36)
	Mood	*n* = 6	*n* = 10	*n* = 1	*n* = 5
		More effective than activity therapy (12)	More effective than usual care (29, 39). On a par with activity sessions (5, 37) or reminiscence (3, 4, 60). Less effective than music sessions (35).	On a par with relaxation therapy, hand massage/ aromatherapy or active therapy (27)	On a par with usual care (36, 48), activity sessions (6*, 36, 48) or music sessions (49)
	Neuropsychiatric symptoms		*n* = 3		
			More effective than activity sessions (48), or on a par with activity sessions (36) or usual care (32)		
	Relaxation	*n* = 8	*n* = 4		*n* = 1
		More effective than usual care (11), massage (11), or activity sessions (12). On a par with a combination of snoezelen and massage (11).	On a par with music sessions (35) or activity sessions (37)		Positive effect of indoor gardening (18)
	Wandering and restlessness		*n* = 1		
			On a par with common best practice (8)		
	Wellbeing				*n* = 1
					On a par with usual care (53)
**Health** **(physical)**	Falls				*n* = 1
					On a par with usual care with visits by volunteers (24)
	Medication			*n* = 1	
				On a par with usual care (13)	
	Standing balance	*n* = 1			*n* = 1
		More effective than usual care or a television condition (57)			On a par with usual care with visits by volunteers (24)
**Participation**	Engagement/interaction with social and physical environment	*n* = 8	*n* = 13	*n* = 4	*n* = 3
		More effective than usual care (16), usual occupational therapy (23), or a living room with low carer attention (19). On a par with a living room with high carer attention (19). Less effective than an outdoor activity (16).	More effective than usual care (29, 32, 39, 53). On a par with reminiscence (3, 4), activity sessions (5, 37), or music sessions (35).	On a par with usual care (38), usual classroom (58), or living room (17, 61). Positive effect of stimulus preference room (17).	On a par with outdoor gardening (1) or activity sessions (6). Positive effect of indoor gardening (18*).
	Family occupations and relations	*n* = 2			
		NA			
**Context**	Discharge rate			*n* = 1	
				On a par with activity sessions (13)	
	Relationship with professionals	*n* = 5	*n* = 4		
		NA	NA		
	Teachers’ knowledge of children	*n* = 1			
		NA			

Note: where applicable, an explanation of the effect in relation to one or more control interventions is provided below the representation of the number of articles; *n* = number of studies; AAIDD = American Association on Intellectual and Developmental Disabilities; NA = not applicable.

⁎ Reported both negative effect and no effect.

The most-reported positive effects in people with intellectual disability were reduced challenging behaviour (*n* = 11, 36.7%), increased relaxation (*n* = 8, 26.7%), and increased engagement with the social and physical environment (*n* = 8, 26.7%). In people with dementia, the most frequently reported positive effects were increased engagement with the social and physical environment (*n* = 13, 40.6%), improved mood (*n* = 10, 31.3%), and reduced agitation (*n* = 8, 25.0%). Negative effects were reported alongside no effects. In the study by Baker et al. (2003), a subgroup of people with dementia were less happy/content after snoezelen than before. A negative influence on mood in some participants was also reported in the qualitative study by Lorusso et al. (2020). The study by Goto et al. (2014) reported participants with dementia who left the snoezelen environment or became less engaged. Lastly, one study involving three people with an intellectual disability reported no effect in two cases and an increase of challenging behaviour in one case (McKee et al., 2007). Of the studies that reported no overall effect, three reported substantial individual differences; snoezelen had a positive effect on some participants (for example, on activity level or relaxation) and no effect on others (Tunson and Candler, 2010; Van Diepen et al., 2002; Vlaskamp et al., 2003). The study by Shapiro et al. (1997), which reported an overall positive effect, also reported individual differences; snoezelen had an activating effect on some participants and a calming effect on others.

Of 20 studies that compared snoezelen with usual care (that is, regular classroom, usual living room), 16 reported that snoezelen was more effective than usual care. When compared with other interventions, however, snoezelen was often still found to be effective, although not always more effective than control interventions (see Table 4). Overall, snoezelen was generally more effective than control interventions for people with intellectual disabilities than for people with dementia. For example, for people with intellectual disabilities, snoezelen was more effective than recreational activities in terms of increasing adaptive behaviour (Shapiro et al., 1997) and improving mental health (S. Chan et al., 2005; Fava and Strauss, 2010; Shapiro et al., 1997). For people with dementia, there was often no difference in effects between snoezelen and recreational activity sessions (for example, Baker et al., 2001; Maseda et al., 2014).

Some outcomes derived primarily from qualitative studies; this mainly concerned relationships with family and professionals. Nine studies reported an improved relationship between healthcare professionals and participants (intellectual disabilities *n* = 5, 16.7%; dementia *n* = 4, 12.5%). For example, in the study by Hope et al. (2004), staff reported that the use of snoezelen allowed them to discover new things about the participant and their personhood. Although no details were provided, it is also reported that snoezelen led to improved responses from health professionals towards participants (Anderson et al., 2011), to professionals feeling more relaxed (Collier and Jakob, 2017; Lorusso et al., 2020), and to changes in care delivery (Hope et al., 2004). Two studies specifically involving people with intellectual disabilities evaluated the effect of snoezelen on family encounters. Both concluded that snoezelen had a positive impact on family occupations and relationships, for example, by experiencing greater intimacy, more close physical contact, and sharing happy moments (Nasser et al., 2004; Sachs and Nasser, 2009).

### 4.7 Participant and application characteristics in relation to effects

Due to limited reported details on the application of snoezelen and the large variation in measured effects, it was not possible to establish a relationship between the effects and characteristics of snoezelen. Based on our analysis and the assumptions of the authors of the studies that were included, characteristics that may be related to effects were identified. An overview containing application characteristics and outcomes in relation to each other is available as supplementary material in S-Table III.

#### 4.7.1 Participant characteristics

Participants who seem to benefit most from snoezelen are those with more severe levels of intellectual disability or dementia (Baillon et al., 2005; Kaplan et al., 2006; Sánchez et al., 2016). In both target groups, the volition and state of mind of participants were discussed as factors of influence (e.g., Baillon et al., 2005). Willingness and cooperation were associated with positive effects (Novakovic et al., 2019), and indifference or dislike with no effects or negative effects (Fava and Strauss, 2010; Goto et al., 2014; Tunson and Candler, 2010; Vlaskamp et al., 2003). It is suggested, though still unclear, that various participant characteristics play a role in effects, such as the type of challenging behaviour a person displays (Kaplan et al., 2006), a diagnosis of autism (Fava and Strauss, 2010), or severe motor and linguistic impairments (Fava and Strauss, 2010).

#### 4.7.2 Types of sensory stimuli

It was not possible to identify the types of stimuli that were used to achieve a certain result and whether this was successful. Some indications were provided; for example, predictable, non-demanding, constant, gentle, or long-lasting stimuli presumably had a positive effect on relaxation (S. Chan et al., 2005; Shapiro et al., 1997). Both non-contingent stimuli (that is, not depending on the participant's behaviour) and contingent stimuli resulted in positive effects (Hill et al., 2012; Staal et al., 2007; Vlaskamp et al., 2003). For the individual senses, music was addressed in six studies as a presumed factor in positive effects (Lindsay et al., 2001; Lorusso et al., 2020; Maseda et al., 2018; Sánchez et al., 2016; Shapiro et al., 1997; Toro, 2019). All four studies that specifically reported the use of vestibular and proprioceptive stimuli to address challenging behaviour reported a reduction in challenging behaviour (Cuvo et al., 2001; Fava and Strauss, 2010; Kaplan et al., 2006; Novakovic et al., 2019).

#### 4.7.3 Frequency and duration

There were similar variations in the frequency and duration of snoezelen in studies showing effects and no effects. For studies showing an effect, the most common frequency was twice a week and the most common duration was 30 min, which is similar to studies showing no effects.

#### 4.7.4 Strategies in applying stimuli

Some strategies may relate to positive effects. Of the 15 studies that reported the use of a session structure, 14 reported a positive effect. In particular, the sensitive introduction of snoezelen, to familiarize the participant and support person with the intervention, was reported as a likely positive factor (Baillon et al., 2005; Staal et al., 2007). Eleven out of 12 studies that used a preference assessment reported positive effects. The ability to control stimuli (Fava and Strauss, 2010) and the possibility of using various stimuli (Maseda et al., 2018; Sánchez et al., 2016) are discussed as positive characteristics. Novelty in the multisensory environment is mentioned as a positive factor in learning (Moir, 2010) and interaction (Cuvo et al., 2001), compared to lack of novelty in usual environments. In one study, snoezelen was deemed boring and no effect was reported (Goto et al., 2014). The effect of the level of structure or directedness during snoezelen is unclear. Eighteen of the 21 studies that used a non-directive approach reported effects; for example, on reducing challenging behaviour (Fava and Strauss, 2010). However, a (semi-)structured and directive approach that included prompting by the support person could also have positive effects, for example, on interaction (Cuvo et al., 2001; Fava and Strauss, 2010).

#### 4.7.5 Social context

Of the 36 studies where snoezelen was applied individually, 31 reported positive effects. Of the nine studies that did not report an effect, five applied snoezelen in a small group. Various studies discussed one-to-one attention from support persons as having a positive effect (for example, Maseda et al., 2014, 2014). The influence of social attention on effects was studied only by Hill et al. (2012), who concluded that it influenced engagement but not challenging behaviour, suggesting that effects derived not only from social attention but also from sensory stimulation (Hill et al., 2012).

#### 4.7.6 Characteristics of support person

Characteristics of the support person that were assumed to have a positive effect were familiarity with the participant (S. Chan et al., 2005), sensitivity towards the participant (Lindsay et al., 1997; Staal et al., 2007), and a positive attitude towards the intervention (Lindsay et al., 1997). Support persons varied in studies with and without effects in terms of their occupation, training, and role during snoezelen.

## 5 Discussion

Snoezelen is applied in a wide variety of target groups, but scientific studies have mainly focused on the application and effects for people with intellectual disabilities and dementia. This review focused on these two target groups. It identified 10 application characteristics that were often only partially described and, if described, showed a great variation in application between studies. Some common features included the use of snoezelen at least twice a week and usually for 30 minutes’ duration, addressing mainly the visual, auditory, and tactile senses. Snoezelen was mostly used individually with a non-directive approach, although with active involvement of the support person to enable snoezelen. Effects were reported across all human functioning dimensions. Positive effects were mostly reported in terms of improved mental health and increased interaction with the social and physical environment. Some effects were inconsistent. It was almost impossible to identify a relationship between application characteristics and effects. Studies that reported the use of a preference assessment, an internal session structure (for example, gradual introduction and winding-down sessions), or individual attention often reported positive effects.

Based on the overall results, snoezelen seems to be more effective than usual care, although the results were inconsistent when compared with other interventions, such as recreational activity sessions. Snoezelen can be relaxing for one person and activating for another. Based on our review, it is unclear in most studies to what extent snoezelen is specifically used to achieve either relaxation or activation and whether these purposes required a specific application. The successful use of preference assessments indicated that an individual approach to applying snoezelen could positively affect outcomes. The lack of knowledge about the role of the support person is conspicuous. The extent to which the target groups in this review needed support suggests that support persons hold a key position when it comes to applying snoezelen. Much is still unclear about how factors relating to the support person (for example, social attention) influence the effects of snoezelen. Furthermore, there are indications that snoezelen also has an effect on support persons and their relationship with the attendee (e.g. Hope et al., 2004; Van Weert et al., 2006; Van Weert, Van Dulmen, Spreeuwenberg, Ribbe, and Bensing, 2005). This implies that snoezelen may have benefits in a broader context, although our understanding of this area is limited.

Snoezelen's presumed relaxing effect seemed to be the most prominent reason for using snoezelen, which is in line with previous findings (Cameron et al., 2020). Theoretical explanations for presumed and established effects are often lacking. Sensory integration theory is incidentally suggested to explain positive outcomes of snoezelen (Kaplan et al., 2006; Novakovic et al., 2019; Shapiro et al., 1997). This theory assumes that everyone has an individual sensory profile that consists of sensory preferences and abilities to process and self-regulate arousal levels (Ayres, 1979). Sensory modulation interventions in line with this theory attracted particular interest in mental health; for example, in managing challenging behaviour (Haig and Hallett, 2023; Scanlan and Novak, 2015; Sutton et al., 2013). In the absence of hypothesized working mechanisms, it is difficult to theorize why snoezelen might result in certain outcomes and therefore be used in an evidence-based manner (Cameron et al., 2020).

Attempts to compare studies on snoezelen have thus far failed in large part due to variation in effects and application and the absence of a theory that incorporates the working mechanisms of snoezelen. Recognizing different types of snoezelen and building a theory on the application of these different types is a first step to better understand what works, for whom, and in what context (Cameron et al., 2020). It would be valuable to investigate the extent to which the application characteristics we have identified meet the needs of individuals within subgroups of people with intellectual disabilities or dementia or the needs of other target groups. Based on our review, we suggest that an intervention description be used that includes the purpose for using snoezelen and the considered use of 10 application characteristics regarding approach, application, context, and conditions. Furthermore, we have provided a first step towards gaining an understanding of the working mechanisms of snoezelen by outlining application characteristics in relation to effects. Given the key role of the support person, we suggest that the further development of an intervention theory and description should include (tacit) knowledge of practitioners to develop a more detailed application framework for snoezelen. Sensory integration theory suggests that it might be valuable to further understand how individual sensory needs and preferences could influence the application of snoezelen. Similarities and contradictions between snoezelen, sensory integration therapy, or other sensory modulation interventions could be explored further. A concept theory on working elements and influencing contextual factors could be further developed by effect studies that manipulate the presumed working elements.

### 5.1 Limitations

In this study, we defined snoezelen as experiencing sensory stimuli in an adapted environment. The characteristic snoezelen environment turned out to be a decisive element in the inclusion of articles, given the variation in intervention names and the frequent lack of a described intervention goal. Our selection approach may have led to the inclusion of interventions that were deliberately not referred to as snoezelen. We may also have missed variations of snoezelen. For example, we are aware that a form of snoezelen is implemented without an adapted environment and could take place in everyday environments, such as during morning care (Van Weert, Van Dulmen, Spreeuwenberg, Ribbe, et al., 2005). In addition, although application characteristics were not always reported in the studies, this does not mean that they were not considered and consciously applied. We focused only on studies conducted on snoezelen and did not therefore include practice-based knowledge. Input from practitioners can further specify characteristics and possibly add details or new characteristics to the ten identified in this study.

The review included studies with various levels of evidence and quality appraisals. Small-scale studies are considered valuable in populations with complex combinations of abilities and disabilities (Maes et al., 2021) and were therefore also considered valuable in this review. However, comparing studies is more difficult when a range of methods are used. By also including studies of low quality, we gained more insight into the application characteristics of snoezelen. However, using studies with an overall moderate quality complicated conclusions about the effectiveness of snoezelen and the relationship between characteristics and effects.

## 6 Conclusions

Although the results are inconclusive, snoezelen can have positive effects in people with intellectual disabilities and dementia, as well as in the people applying the interventions. To apply snoezelen purposefully and effectively, we need to further understand what snoezelen's working mechanisms are and for whom and in what context a specific use of snoezelen applies. This study identified 10 application characteristics of snoezelen and made a first step toward describing its potential working mechanisms. Also incorporating the perspective of practitioners can further develop an intervention theory and application framework for snoezelen.

## Funding sources

This study was conducted with funding from ZonMw (the Netherlands Organisation for Health Research and Development) (No. 641001104).

## CRediT authorship contribution statement

**Gemma Testerink:** Conceptualization, Methodology, Formal analysis, Writing – original draft. **Annet ten Brug:** Conceptualization, Supervision, Writing – original draft, Writing – review & editing. **Gerdine Douma:** Conceptualization, Supervision, Writing – review & editing. **Annette van der Putten:** Supervision, Writing – review & editing, Funding acquisition.

## Declaration of Competing Interest

The authors declare that they have no known competing financial interests or personal relationships that could have appeared to influence the work reported in this paper.

## Appendix Supplementary materials

Image, application 1
