# Supplementary material for: Snoezelen in people with intellectual disability or dementia: A systematic review
Source: Int J Nurs Stud Adv. 2023 Aug 25;5:100152. doi: 10.1016/j.ijnsa.2023.100152 (PMC11080487; doi:10.1016/j.ijnsa.2023.100152)
Supplement: Supplementary file 1 [file mmc1.pdf]

# Supplementary material

*S-Table I*

*Snoezelen in target groups other than people with intellectual disabilities or dementia (*n *= 24)*

| **Target group (↓)** | **Author, year**<br><br>**Country** | **Design**<br><br>**Control condition** | **Participants**<br>**1) *n* (age)**<br>**2) female/male**<br>**3) diagnosis**<br>**4) setting** | **Study aim** | **Snoezelen**<br>**1) goal**<br>**2) individual/group**<br>**3) frequency**<br>**4) duration**<br>**5) approach/strategies used** | **Data collection** | **Results** |
|---|---|---|---|---|---|---|---|
| Brain injury | Gómez et al., 2016<br><br>Spain | Non-randomized study<br><br>Control: healthy individuals | 1) *n* = 36 (TBI *M* = 38.06, *SD* 8.25; CP *M* = 44.61, *SD* 10.89)<br>2) TBI 3F, 15M; CP 7F, 11M<br>3) cerebral palsy (CP), traumatic brain injury (TBI)<br>4) unclear | Assess changes induced in electroencephalographic (EEG) activity | 1) relaxing but also stimulating<br>2) individually<br>3) one session<br>4) 18 min<br>5) therapist involved in facilitating interaction. Stimuli provided in four parts. | EEG recording | Effect:<br>↓ oscillatory activity<br>↓ EEG complexity and irregularity<br><br>Alterations implying ↑ relaxation |
| | Hotz et al., 2006<br><br>United States | Non-randomized study; case series<br><br>No control | 1) *n* = 15 (*M* = 9.87 yrs; range 1.2-16.9)<br>2) 4F, 11M<br>3) severe TBI, anoxic event<br>4) paediatric rehabilitation unit | Investigate the effects on physiological, cognitive and behavioural changes | 1) depending on level of arousal or relaxation<br>2) individually<br>3) 3x per week<br>4) 30-40 min<br>5) using one-to-three pieces of equipment in a sequential order, facilitating physical contact and interaction, not interact with subject's choice or pace, enabling approach, according to protocol. | Glasgow Coma Scale; physiological measures; Ranchos Los Amigos Scale; Agitated Behaviour Scale; Functional Independent Measure | Effect:<br>↓ heart rate<br>↓ muscle tone<br>↓ agitation<br>↑ overall cognitive outcome<br><br>No effect on blood pressure |

| | | | | | | |
|---|---|---|---|---|---|---|
| Lehrer, et al., 2022<br><br>Israel | Non-randomized study<br><br>No control | 1) *n* = 35 (UWS *M* = 46.7 yrs, *SD* = 17.3, range 21-69; MCS *M* = 51.6, *SD* = 13.9, range 21-83)<br>2) 7F, 28M<br>3) unresponsive wakefulness syndrome (UWS) or minimally conscious state (MCS)<br>4) rehabilitation medical centre | Explore effect on responsiveness through physiological and clinical effects | 1) NR<br>2) unclear<br>3) one session<br>4) NR<br>5) 15 stimuli (3 for each sense) applied in a constant order for 1 minute with 5 minutes rest in between. | Loewenstein communication scale; transcranial Doppler monitoring cerebral blood flow velocity; heart rate | Effect in MCS group:<br>↑ heart rate<br>↓ cerebral blood flow<br>↑ communication<br><br>Effect in UWS group:<br>- Minor physiological effect |
| Poza, Gómez, Gutiérrez, Mendoza, & Hornero, 2013<br><br>Spain | Non-randomized study<br><br>Control: healthy individuals | 1) *n* = 18 (*M* = 38.4 yrs, *SD* = 5.1, range 29-46)<br>2) 7F, 11M<br>3) mild to severe brain injury<br>4) national reference centre for people with severe disabilities | Describe the changes induced in the EEG brain oscillations | 1) relaxing but also stimulating<br>2) individually<br>3) one session<br>4) 18 min<br>5) therapist involved in facilitating interaction. Stimuli provided in four parts. | EEG recording | Effect:<br>- Slowing of EEG oscillatory activity, suggesting a reflection of the state of relaxation. |
| Priyadharsini & Mukil Singh, 2016<br><br>India | Non-randomized study<br><br>No control | 1) *n* = 12 (range 3-13 yrs)<br>2) both male and female children<br>3) cerebral palsy<br>4) national special school | Assess effect on reduction of maladaptive behaviour | 1) NR<br>2) individually<br>3) 3x per week<br>4) NR<br>5) visual, auditory, tactile and olfactory modalities in a specially arranged room. | Standardized behavioural assessment scale for Indian children with cerebral palsy | Effect:<br>↓ maladaptive behaviour |
| Yang, Zhang, Zhao, & Chen, 2021<br><br>China | RCT<br><br>Control: usual rehabilitation therapy | 1) *n* = 84 (range 3 to 24 months)<br>2) 22F, 62M<br>3) global developmental delay; children too young to complete a test on intellectual functioning<br>4) hospital rehabilitation department | Study effect on improving rehabilitation and reduce the incidence of disability | 1) NR<br>2) NR<br>3) NR<br>4) NR<br>5) NR | Gesell Developmental Observation (five domains: developmental; letter/numbers; language/ comprehension; visual/ spatial; and social-emotional/ adaptive) | Effect:<br>↑ cognitive, language, motor and social-emotional responses<br><br>Effect in both groups, though significantly higher in intervention group. |

# Supplementary material - Snoezelen: A systematic review

| | | | | | | | |
|---|---|---|---|---|---|---|---|
| Chronic pain | Schofield & Davis, 2000; Schofield, Davies, & Hutchinson, 1998a, 1998b<br><br>United Kingdom | Mixed method design; RCT and qualitative study<br><br>Control: usual relaxation approach | 1) *n* = 73 (experimental group *M* = 48.2 yrs; control group *M* = 48 yrs)<br>2) 43F, 30M<br>3) chronic pain<br>4) hospital | Compare the use of snoezelen as a protentional relaxation and distraction strategy | 1) relaxation<br>2) NR<br>3) 2 sessions<br>4) 3 hours<br>5) NR | Intensity and quality of pain using the Visual Analogue Scale; McGill Pain Questionnaire; Sickness Impact Scale; Self Efficacy Questionnaire; Coping Strategies Questionnaire; semi-structured interviews | Effect:<br>↓ pain<br>↑ self-efficacy<br>↑ coping strategies<br>↑ coping self-statements<br>↓ level of disability<br><br>Reductions observed for anxiety, depression, and self-efficacy for both groups, though not significant. |
| Dental health | Shapiro, Melmed, Sgan-Cohen, Eli, & Parush, 2007 | RCT; cross over design<br><br>Control: conventional dental environment | 1) *n* = 19 (*M* = 8.8 yrs, *SD* = 1.74, range 6-11 yrs)<br>2) 6F, 13M<br>3) need for manual dental scaling and tooth cleaning<br>4) special needs dental clinic | Assess the efficacy in reducing anxiety among children undergoing scaling and polishing by a dental hygienist | 1) NR<br>2) individually<br>3) one session per condition with 4 months in between<br>4) 20-25 min<br>5) adapted visual sensation (for example, no overhead fluorescent lighting), added auditory and somato-sensory stimuli, deep pressure through a regular dental X-ray vest | Anxiety and Cooperation Scale; questionnaire for parents on anxiety; Negative Dental Behaviours Checklist; Electrodermal activity to measure relaxation and arousal | Effect compared to control<br>↑ relaxation<br><br>80% percent of the children indicated that they preferred the sensory-adapted dental environment |
| Huntington's disease | Leng et al., 2003<br><br>United Kingdom | RCT<br><br>Control: relaxation activities | 1) *n* = 12 (age NR)<br>2) NR<br>3) mid-late stage Huntington's disease<br>4) specialist residential unit | Investigate whether behavioural, motor and physiological responses to a multisensory environment are effective as therapeutic or leisure activity | 1) NR<br>2) individually<br>3) 2x per week<br>4) 30 min<br>5) stimuli presented in a nonsequential unpatterned manner. All senses, except taste. Non-directive enabling approach. Suited to individual preferences. | Rehabilitation evaluation – Hall and Baker; Behaviour and Mood Disturbance Scale; dyskinesia section of the St Hans Rating Scale; physiological measures; Interact | Effect compared to control<br>↑ mood<br><br>No effect on behaviour, involuntary movements or physiological measures. |
| Labour/ giving birth | Hauck, Rivers & Doherty, 2008<br><br>Australia | Qualitative study; grounded theory-based analysis | 1) *n* = 16 (*M* = 27.6 yrs; range 19-36)<br>2) 16F<br>3) women in labour<br>4) hospital | Explore women's experience of using snoezelen during labour | 1) NR<br>2) individually<br>3) one session<br>4) mean 2 hours, mode 1 hour<br>5) a midwifery-led initiative, room specifically designed for maternity clients | In-depth interview | Perceived benefits of what snoezelen can offer:<br>↓ distraction<br>↑ relaxation<br>↑ comfort<br>↑ environmental control<br>- choice of complementary therapies<br>- safety in a home-like atmosphere |

| | | | | | | | |
|---|---|---|---|---|---|---|---|
| | Manesh, Kalati, & Hosseini, 2015<br><br>Iran | RCT<br><br>Control: usual care | 1) *n* = 100 (experimental group *M* = 22 yrs, *SD* 4.22; control group *M* = 25 yrs, *SD* = 4.71)<br>2) 100F<br>3) women in labour<br>4) labour unit | Evaluate effects on childbearing outcomes | 1) NR<br>2) individually<br>3) one session<br>4) NR<br>5) women could walk, sit, lie down based on their wishes. Stimuli available for all senses | Pain intensity using the Visual Analogue Scale; duration of labour; perinea status | Effect compared to control:<br>- less increased intensity of pain |
| | Momeni, Jamshidimanesh, & Ranjbar, 2020<br><br>Iran | RCT<br><br>Control: usual care | 1) *n* = 130 (intervention group *M* = 26.69 yrs, *SD* = 5.11; control group *M* = 28.25 yrs, *SD* = 4.24)<br>2) 130F<br>3) women in labour<br>4) hospital | Evaluate effects on fear, anxiety, and satisfaction of childbirth | 1) NR<br>2) individually<br>3) one session<br>4) NR<br>5) NR | Harman's Childbirth Attitude questionnaire; Mackey Childbirth Satisfaction Rating scale; Visual Analogue Scale | Effect compared to control:<br>↓ fear in the active phase and postpartum<br>↓ anxiety<br>↑ satisfaction of childbirth |
| | Nielsen & Overgaard, 2020<br><br>Denmark | Qualitative study; hermeneutical-phenomenological method | 1) *n* = 14 (range 24-31 yrs)<br>2) 14F<br>3) women in labour<br>4) regional hospital | Explore women's experience of the environment and its ability to support the concept of patient-centredness | 1) promoting feelings of well-being, freedom and control<br>2) individually<br>3) one session<br>4) NR<br>5) visual and auditory stimuli providing distraction, furniture resembling home environment, dimmed light. | Semi-structured interviews | Perceived benefits<br>- Emotional support (feeling welcome, midwife and room inseparability)<br>- Involvement of partner (feeling equal, a space for the partner)<br>- Physical comfort (positive distractions, capturing the room) |
| Mental health | Cheng, Hsu, Shen, Hsu, & Lin, 2017<br><br>Taiwan | RCT; crossover<br><br>Control: usual care | 1) *n* = 60 (*M* = 42.28 yrs; range 22-64)<br>2) 33F, 27M<br>3) chronic schizophrenia<br>4) psychiatric hospital | Evaluate the effectiveness and assess the correlation between intervention dose and effectiveness | 1) NR<br>2) unclear<br>3) 2x per week<br>4) 30 min<br>5) participants chose their own stimulus after entering the multisensory room. Observer assisted when needed. | Brief Psychiatric Rating Scale; Hospital Anxiety and Depression Scale; Schizophrenia Patients Subjective Well-being; NeXus-4 for physiological responses | Effect:<br>↓ negative emotional reactions<br>↓ subjective anxiety level<br>Stabilized respiratory and heart rates<br><br>No effect on psychotic symptoms and wellbeing<br><br>Positive correlation between efficacy of treatment and frequency of the intervention |

| | | | | | | |
|---|---|---|---|---|---|---|
| Collier, Staal & Homel, 2018<br><br>United Kingdom, United States of America | RCT<br><br>Control: hospital/ university unit lounge | 1) *n* = 16 (M = 42.75 yrs; range 24-61)<br>2) 16F<br>3) NA, mental health nurses<br>4) acute care psychiatric inpatient unit in a hospital or mental health services | Evaluate whether snoezelen reduced occupational stress | 1) NR<br>2) individually<br>3) 2x per week<br>4) 30 min<br>5) treatment and room set up based on preference assessment | State Trait Anxiety Inventory; Profile of Mood States; physiologic measurement of pulse; satisfaction questionnaire | Effect:<br>↓ pulse rate<br>↓ anxiety<br>↑ mood<br>↑ satisfaction |
| Di Taranto et al., 2022<br><br>Italy | Non-randomized study; case study | 1) *n* = 1 (19 yrs)<br>2) 1F<br>3) alexithymia (difficulty in identification and verbal expression of emotions)<br>4) family meeting in psychiatric ward | Illustrate effect on emotional openness for the start of a psychotherapeutic pathway | 1) NR<br>2) individually<br>3) 2x per week<br>4) 45 min<br>5) visual, auditory, and olfactory stimulations. Structured order with equipment. | Minnesota Multiphasic Personality Inventory-2; Brief Psychiatric Rating Scale; Hamilton Anxiety Scale; Hamilton Rating Scale for Depression; Toronto Alexithymia Scale; Toronto Structured Interview for Alexithymia | Effect:<br>↓ symptoms of alexithymia<br>↑ emotional openness |
| Kim & Park, 2022<br><br>South Korea | Non-randomized study; multiple treatment design<br><br>Control: multipurpose room | 1) *n* = 3 (3, 12, 4 yrs)<br>2) 1F, 2M<br>3) autism and hypo-visual sensitivity, mixed auditory sensitivity, and/or hypo-auditory sensitivity<br>4) children's developmental centre | Investigate influence on the target behaviours | 1) relaxation or stimulation tailored to particular sensory characteristics<br>2) individually<br>3) 3x per week<br>4) NR<br>5) based on the sensory profiles intervention focused on visual and auditory stimuli with 20 selected items. | Short sensory profile; sensory characteristics background; selected target behaviours (sucking fingers, glancing sideways, flapping hands, making nonspeech sounds, sucking thumb or mouthing material) | Effect:<br>↓ selected target behaviours |
| Nelson & Hummel, 2022<br><br>United States | RCT<br><br>Control: recreational therapy using table games | 1) *n* = 35 (*M* = 35 yrs, *SD* = 13.16, range 19-58)<br>2) 11F, 24M<br>3) substance use disorder<br>4) residential treatment facility | Examine the effect on anxiety, agitation, and pain | 1) NR<br>2) Individually<br>3) 2x per week<br>4) 30-45 min<br>5) introduction of four activities after which the participant could choose based on their preference (breathing with bubbles, tactile transfer, comb away your cares, and savour the moment) | Brief Agitation Measure; Hamilton Anxiety Rating Scale; Visual Analog Scale for Pain; heart rate; respiration rate; participant survey | Effect compared to control:<br>↓ anxiety<br>↓ agitation<br>↓ pain<br>↓ respiration rate<br><br>No effect on heart rate.<br><br>All participants reported multisensory intervention as a valuable addition to their treatment. |

| | | | | | | |
|---|---|---|---|---|---|---|
| Reddon, Hoang, Sehgal, & Marjanovic, 2004<br><br>Canada | Non-randomized study<br><br>Control: hospital employees | 1) patients *n* = 50 (female *M* = 44.12, *SD* = 11.47; male *M* = 43.60, *SD* = 12.36). Employees *n* = 50 (female *M* = 40.36, *SD* = 9.12; male *M* = 38.64, *SD* = 9.58)<br>2) patients 25F, 25M; employees 25F, 25M<br>3) psychiatric patients<br>4) hospital | Evaluate effect on patients and hospital employees | 1) gentle stimulation<br>2) individually<br>3) one session<br>4) 20 min<br>5) participants indicated what equipment or modalities they were interested in using. Stimuli were systematically introduced. Once all were introduced, there was no more interruption and/ or interference by investigators. | Heart rate and percentage of blood oxygen levels; Galvanic Skin Response; questionnaire on psychological benefits | Effect, but no difference from control group:<br>↓ heart rate<br>↓ Galvanic Skin Response<br><br>Psychological effects in both groups: sleepier, passive, relaxed, cheerful, focused, optimistic, calm, and comfortable<br><br>No effect on blood oxygen levels. |
| Unwin, Powell, & Jones, 2021<br><br>United Kingdom | Mixed method: qualitative study and quantitative descriptive study | Study 1<br>1) *n* = 10 (*M* = 44.3 yrs, *SD* = 14.0, range 24-62)<br>2) 9F, 1M<br><br>Study 2<br>1) *n* = 102 (*M* = 40.4 yrs, *SD* = 10.7, range 21-68)<br>2) 93F, 9M<br><br>Study 1 & 2<br>3) NA (educational practitioners working with children with autism)<br>4) special needs schools | Gain insight into the beliefs and experiences of practitioners about possible outcomes and overall efficacy, and the factors that may affect possible outcomes | NA | Study 1: interviews<br>Study 2: survey | Perceived benefits:<br>↑ focus and attention<br>↑ mood<br>↑ social interaction and communication<br>↓ repetitive motor behaviours<br>↓ anxiety |
| Unwin, Powell, & Jones, 2022<br><br>United Kingdom | Non-randomized study; counter-balanced<br><br>2 snoezelen conditions: active-change and passive-change | 1) *n* = 41 (*M* = 8 yrs, *SD* = 2.05, range 4-12)<br>2) 8F, 33M<br>3) children with autism, the mean intelligence quotient is average (range between 'intellectual disability' and 'gifted')<br>4) NR | To evaluate whether having control of multisensory equipment affects behaviour and arousal compared to not having control | 1) NR<br>2) individually<br>3) one session<br>4) 15min per condition, with<br>5) In the Active-Change condition, participants changed sensory aspects themselves using an iPad or by directly pressing the equipment. In the Passive-Change condition, most stimuli changed every 3 seconds without the participants input. | Video observation and coding of: repetitive motor behaviours; sensory behaviours; social interaction and communication; anxiety; positive affect; attention | Having control was associated with:<br>↑ attention<br>↓ repetitive motor behaviours<br>↓ sensory behaviours<br>↓ activity levels<br>↓ stereotyped speech and vocalisations<br><br>Not affected by condition were social behaviour, anxiety, positive affect and arousal. |

| | | | | | | | |
|---|---|---|---|---|---|---|---|
| Motor dysfunction | Lavie, Shapiro, & Julius, 2005<br><br>Israel | Non-randomized study: case study<br><br>No control | 1) *n* = 1 (19 yrs)<br>2) 1F<br>3) cerebellar vestibular dysfunction<br>4) therapeutic swimming and recreation centre | Effect of combining hydrotherapy and snoezelen | 1) to help achieve optimal arousal levels<br>2) individually<br>3) 1x per week<br>4) NR<br>5) stimuli are manipulated and provided according to needs of individual | Observations | Perceived benefits on psychological, physical and social skills. |
| Palliative care | Schofield, 2009<br><br>United Kingdom | RCT<br><br>Control: usual 'quiet room' setting | 1) *n* = 26<br>2) NR<br>3) patients with moderate levels of anxiety attending palliative day care<br>4) hospice day unit | Explore the feasibility of using snoezelen | 1) NR<br>2) individually<br>3) 2 sessions<br>4) 60 min<br>5) patients spent time in the room alone, but were able to call staff | Hospital Anxiety and Depression scale; European Organisation for the Research and Treatment of Cancer Core 30 questionnaire; semi-structured interview | Effect compared to control:<br>↓ anxiety<br><br>No effect on quality of life.<br><br>Perceived benefit:<br>↑ relaxation |

*Note:* n *= number of participants; RCT = randomized controlled trial;* M *= mean;* SD *= standard deviation; F = female; M = male; NR = not reported; NA = not applicable; TBI = traumatic brain injury; CP = cerebral palsy; EEG = electroencephalographic; UWS = unresponsive wakefulness syndrome; MCS = minimally conscious state;*

*S-Table II*
*Articles per application characteristic (the reference numbers in this table correspond to the reference numbers in Table 2 of the main article)*

| | | ID reference number | *n* | DM reference number | *n* |
|---|---|---|---|---|---|
| **Senses addressed** | Visual | 2, 11, 16, 17, 22, 23, 26, 27, 28, 44, 50, 51, 56, 57, 59, 61 | **16** | 1, 5, 6, 14, 24, 29, 30, 35, 36, 45, 48, 49 | **12** |
| | Auditory | 2, 11, 16, 17, 22, 23, 26, 27, 28, 44, 50, 51, 56, 57, 59, 61 | **16** | 1, 5, 6, 14, 29, 30, 35, 36, 45, 48, 49 | **11** |
| | Tactile | 2, 11, 16, 17, 22, 23, 26, 27, 28, 44, 50, 51, 56, 57, 59, 61 | **16** | 1, 5, 6, 14, 24, 29, 35, 36, 45, 48, 49 | **11** |
| | Olfactory | 2, 11, 16, 17, 26, 27, 44, 50, 51, 56, 57, 61 | **12** | 1, 5, 6, 14, 29, 30, 35, 36, 45, 48, 49 | **11** |
| | Gustatory | 2 | **1** | 14 | **1** |
| | Proprioception | 23, 28, 44 | **3** | 14, 24 | **2** |
| | Vestibular | 16, 17, 23, 44 | **4** | 14, 45 | **2** |
| | NR/unclear | 10, 12, 13, 19, 25, 31, 34, 38, 42, 43, 46, 52, 55, 58 | **14** | 3, 4, 7, 8, 9, 15, 18, 20, 21, 32, 33, 37, 39, 40, 41, 47, 53, 54, 60, 62 | **20** |
| **Materials and equipment used** | Music player | 2, 10, 16, 17, 19, 22, 23, 26, 27, 31, 34, 38, 42, 43, 44, 50, 51, 52, 55, 56, 57, 58, 61 | **24** | 1, 6, 7, 14, 15, 18, 20, 24, 29, 30, 32, 33, 35, 36, 37, 39, 40, 41, 45, 47, 48, 49, 53, 60 | **24** |
| | Bubble tube | 2, 10, 12, 16, 17, 19, 22, 23, 25, 26, 27, 31, 34, 38, 42, 44, 46, 52, 55, 56, 58 | **21** | 1, 6, 7, 9, 14, 15, 18, 20, 29, 30, 35, 36, 37, 39, 40, 48, 49, 53, 60, 62 | **20** |

# Supplementary material - Snoezelen: A systematic review

| | | | | | |
|---|---|---|---|---|---|
| | Aromatics | 2, 11, 12, 16, 17, 25, 26, 27, 38, 44, 46, 50, 51, 55, 56, 57, 58 | **17** | 1, 6, 7, 14, 15, 18, 24, 29, 30, 33, 35, 36, 37, 40, 41, 45, 47, 48, 49, 53, 60, 62 | **22** |
| | Projection equipment | 2, 10, 11, 12, 16, 17, 22, 23, 25, 26, 27, 28, 38, 42, 44, 46, 50, 51, 52, 55, 56 | **21** | 1, 6, 7, 14, 18, 20, 24, 29, 30, 33, 35, 36, 37, 39, 45, 47, 48, 49, 53, 60 | **20** |
| | Fibre optic material | 2, 10, 11, 17, 22, 23, 25, 26, 27, 34, 38, 42, 44, 51, 52, 56 | **16** | 1, 6, 7, 9, 14, 15, 18, 20, 29, 30, 32, 35, 36, 37, 39, 48, 49, 53, 60, 62 | **20** |
| | Mirroring material | 2, 11, 12, 19, 22, 25, 26, 27, 31, 42, 44, 51, 56, | **13** | 1, 7, 20, 35, 36, 37, 40, 48, 49, 60 | **10** |
| | Tactile material | 2, 10, 12, 16, 17, 25, 26, 27, 50, 52, 56, | **11** | 1, 6, 7, 9, 14, 18, 20, 32, 35, 36, 37, 39, 45, 48, 49 | **15** |
| | (Water)bed/ mattress | 22, 28, 31, 34, 44, 46, 50, 51, 52, 56, 57, 61 | **12** | 7, 14, 33, 35, 36, 37, 47, 48, 49 | **9** |
| | Vibrating material | 2, 11, 16, 17, 44, 50, 51, 57 | **8** | 1, 7, 14, 24, 30, 36, 37, 40, 62 | **9** |
| | Bean bag | 2, 12, 16, 17, 23, 25, 34, 38, 51, 52 | **10** | 14, 40 | **2** |
| | NR | 13, 59 | **2** | 3, 4, 5, 8, 21, 54 | **6** |
| **Frequency** | Frequency ≤ 2x week | 10, 11, 19, 23, 25, 28, 31, 34, 57, 59, 61 | **11** | 1, 3, 4, 5, 6, 8, 18, 24, 29, 32, 35, 36, 37, 39, 48, 49, 60 | **17** |
| | Frequency ≥ 3x week | 10, 12, 13, 16, 17, 38, 44, 51, 52, 58 | **10** | 9, 15, 39 | **3** |
| | NR/unclear | 2, 22, 26, 27, 42, 43, 46, 50, 55, 56 | **10** | 7, 14, 20, 21, 30, 33, 40, 41, 45, 47, 53, 54, 62 | **13** |
| **Duration** | Duration ≤ 30 min | 2, 11, 16, 17, 19, 23, 26, 27, 31, 42, 44, 50, 52, 56, 57, 58, 59, 61 | **18** | 1, 5, 6, 9, 18, 20, 24, 32, 35, 36, 37, 39, 41, 48, 49, 53, 54, 62 | **18** |
| | Duration > 30 min | 2, 12, 13, 16, 22, 25, 28, 34, 38, 43, 51 | **11** | 3, 4, 20, 33, 47, 60 | **6** |
| | NR/unclear | 10, 46, 55, | **3** | 7, 8, 14, 15, 21, 29, 30, 40, 45 | **9** |
| **Strategies** | Preference assessment | 31 | **1** | 4, 6, 15, 29, 32, 35, 36, 37, 48, 49, 54 | **11** |
| | Non-directive approach | 11, 12, 13, 16, 17, 31, 38, 44, 52, 57 | **10** | 5, 6, 32, 35, 36, 37, 47, 48, 49, 53, 60 | **11** |
| | Internal session structure | 16, 17, 43, 44 | **4** | 3, 4, 5, 6, 15, 20, 29, 35, 36, 37, 49 | **11** |
| | Unpatterned stimuli | - | **-** | 4, 5, 6, 35, 36, 37, 48, 49 | **8** |
| | No demands | 44 | **1** | 5, 6, 35, 36, 37, 48, 49 | **7** |
| | Other strategies | 2, 19, 23, 28, 42, 55, 56, 61 | **8** | 4, 6, 15, 24, 32, 45, 54 | **7** |
| | NR | 10, 22, 25, 26, 27, 34, 46, 50, 51, 58, 59 | **11** | 1, 7, 8, 9, 14, 18, 21, 30, 33, 39, 40, 41, 62 | **13** |
| **Physical aspects of the MSE** | Size | 2, 12, 16, 17, 19, 25, 34, 38, 44, 51, 52, 59 | **12** | 1, 18, 41 | **3** |
| | White room | 2, 16, 17, 34, 38, 44, 46, 50, 56 | **9** | 53 | **1** |
| | Padding | 2, 19, 25, 38, 44, 46, 50 | **7** | - | **-** |
| | No daylight | 2, 26, 27, 44, 50, 56 | **6** | 18 | **1** |
| | NR/unclear | 10, 11, 13, 22, 23, 28, 31, 42, 43, 55, 57, 58, 61 | **13** | 3, 4, 5, 6, 7, 8, 9, 14, 15, 20, 21, 24, 29, 30, 32, 33, 35, 36, 37, 39, 40, 45, 47, 48, 49, 54, 60, 62 | **28** |
| **Social context in the MSE** | Individual session | 2, 16, 17, 19, 23, 26, 27, 28, 31, 38, 42, 50, 52, 57, 59 | **15** | 1, 3, 4, 5, 6, 7, 18, 20, 24, 29, 32, 35, 36, 37, 39, 48, 49, 53, 54, 60, 62 | **21** |
| | Group session | 2, 12, 13, 22, 34, 43, 44, 46, 51, 58, 61 | **11** | 7, 9 | **2** |
| | NR/unclear | 10, 11, 25, 55, 56 | **5** | 8, 14, 15, 21, 30, 33, 40, 41, 45, 47 | **8** |
| **Type of support person** | Therapist | 22, 23, 28, 43, 44, 46 | **6** | 5, 6, 7, 8, 20, 30, 32, 35, 36, 37, 48, 49, 53, 60 | **14** |
| | Carer/nurse | 11, 12, 13, 17, 19, 31, 38 | **7** | 1, 5, 6, 7, 20, 29, 30, 40, 41, 45 | **10** |

| | | | | | |
|---|---|---|---|---|---|
| | Teacher | <u>2</u>, 22, <u>55</u>, <u>56</u>, 58, 59 | **6** | - | **-** |
| | Other | <u>2</u>, 16, 25, 43, 52, 57 | **6** | 1, <u>14</u>, <u>30</u>, 62 | **4** |
| | NR/unclear | <u>10</u>, 26, 27, 34, 42, 50, 51, 61 | **8** | 3, 4, 9, 15, 18, <u>21</u>, 24, 33, 39, 47, 54 | **11** |
| **Training of support person** | (Some) details | <u>10</u>, 31, 44, <u>55</u>, | **4** | 1, <u>14</u>, 20, <u>30</u>, 40 | **5** |
| | No details | 26, 27, 38 | **3** | 5, 6, 8, 9, 35, 36, 37, 39, 49 | **9** |
| | NR/unclear | <u>2</u>, 11, 12, 13, 16, 17, 19, 22, 23, 25, 28, 34, 42, 43, 46, 50, 51, 52, <u>56</u>, 57, 58, 59, 61 | **23** | 3, 4, <u>7</u>, 15, 18, <u>21</u>, 24, 29, <u>30</u>, 33, 41, 45, 47, 48, 53, 54, 60, 62 | **18** |
| **Role of support person** | Active | <u>2</u>, 11, 13, 16, 17, 19, 23, 28, 31, 34, 38, 42, 43, 44, <u>55</u>, <u>56</u>, 59, 61 | **18** | 1, 9, 24, 32, 35, 36, 37, 39, 49, 60 | **10** |
| | Passive | 12, 16, 17, 31, 44, 52, <u>55</u>, 57 | **8** | 9, 32, 45 | **3** |
| | NR/unclear | <u>10</u>, 22, 25, 26, 27, 46, 50, 51, 58 | **9** | 3, 4, 5, 6, <u>7</u>, 8, <u>14</u>, 15, 18, 20, <u>21</u>, 29, <u>30</u>, 33, 40, 41, 47, 48, 53, 54, 62 | **21** |

*Note:* n *= number of articles; ID = intellectual disability; DM = dementia; NR = not reported; MSE = multisensory environment; underscore = (semi-)open query*

*S-Table III*
*Application characteristics and outcomes in relation to each other*

| | | **Effect** | | | | **No effect** | | | |
|---|---|---|---|---|---|---|---|---|---|
| | **Characteristic** | **ID (*n* = 21) (number of articles reporting)** | ***n*** | **DM (*n* = 24) (number of articles reporting)** | ***n*** | **ID (*n* = 9) (number of articles reporting)** | ***n*** | **DM (*n* = 9) (number of articles reporting)** | ***n*** |
| **Participants** | **Age** | 2 to 74 years | **20** | 54 to 102 years | **22** | 3 to ≥ 71 years | **9** | 68 to 102 years | **9** |
| | **Level or severity of disability** | Mild (*n* = 3); moderate (*n* = 7); severe (*n* = 13); profound (*n* = 13) | **21** | Mild (*n* = 4); moderate (*n* = 11); severe (*n* = 16); very severe (*n* = 3) | **17** | Mild (*n* = 2); moderate (*n* = 3); severe (*n* = 5); profound (*n* = 5) | **9** | Mild (*n* = 2); moderate (*n* = 5); severe (*n* = 8); profound (*n* = 2) | **8** |
| | **Additional impairments** | Psychiatric (*n* = 11); motor (*n* = 10); medical (*n* = 7); sensory (*n* = 5); NR/U (*n* = 3) | **18** | Psychiatric (*n* = 3); motor (*n* = 4); medical (*n* = 2); sensory (*n* = 2); NR/U (*n* = 18) | **6** | Psychiatric (*n* = 4); motor (*n* = 4); medical (*n* = 2); sensory (*n* = 1); NR/U (*n* = 2) | **7** | Psychiatric (*n* = 2); motor (*n* = 1); medical (*n* = 1); sensory (*n* = 0); NR/U (*n* = 6) | **3** |
| | **Setting** | Residential (*n* = 13); school (*n* = 3); day care (*n* = 3); NR/U (*n* = 3) | **18** | Residential (*n* = 20); school (*n* = 0); day care (*n* = 5); NR/U (*n* = 2) | **22** | Residential (*n* = 6); school (*n* = 1); day care (*n* = 1); NR/U (*n* = 1) | **8** | Residential (*n* = 9); school (*n* = 0); day care (*n* = 1); NR/U (*n* = 0) | **9** |
| **Application of stimuli** | **Senses addressed** | Visual (*n* = 13); auditory (*n* = 13); tactile (*n* = 13); olfactory (*n* = 9); gustatory (*n* = 0); proprioception (*n* = 3); vestibular (*n* = 4); NR/U (*n* = 8) | **13** | Visual (*n* = 8); auditory (*n* = 8); tactile (*n* = 8); olfactory (*n* = 8); gustatory (*n* = 0); proprioception (*n* = 0); vestibular (*n* = 1); NR/U (*n* = 16) | **8** | Visual (*n* = 4); auditory (*n* = 4); tactile (*n* = 4); olfactory (*n* = 4); gustatory (*n* = 0); proprioception (*n* = 0); vestibular (*n* = 1); NR/U (*n* = 5) | **4** | Visual (*n* = 6); auditory (*n* = 5); tactile (*n* = 6); olfactory (*n* = 5); gustatory (*n* = 0); proprioception (*n* = 1); vestibular (*n* = 0); NR/U (*n* = 3) | **6** |

| | | | | | | | | | |
|---|---|---|---|---|---|---|---|---|---|
| | **Materials and equipment used** | Music player (*n* = 16); bubble tube (*n* = 14); aromatics (*n* = 12) projection equipment (*n* = 16); fibre optic material (*n* = 11); mirroring material (*n* = 11); tactile materials (*n* = 8); NR/U (*n* = 1) | **20** | Music player (*n* = 18); bubble tube (*n* = 14); aromatics (*n* = 16) projection equipment (*n* = 14); fibre optic material (*n* = 14); mirroring material (*n* = 9); tactile materials (*n* = 10); NR/U (*n* = 5) | **19** | Music player (n = 6); bubble tube (n = 6); aromatics (n = 6) projection equipment (n = 5); fibre optic material (n = 5); mirroring material (n = 3); tactile materials (n = 3); NR/U (*n* = 1) | **8** | Music player (n = 8); bubble tube (n = 8); aromatics (n = 8) projection equipment (n = 8); fibre optic material (n = 8); mirroring material (n = 4); tactile materials (n = 7); NR/U (*n* = 0) | **9** |
| | **Frequency of session(s)** | Range 1 to 7x a week<br>≤ 2x week (*n* = 8)<br>≥ 3x week (*n* = 6)<br>NR/U (*n* = 7) | **14** | Range 1 to 3x a week<br>≤ 2x week (*n* = 14)<br>≥ 3x week (*n* = 2)<br>NR/U (*n* = 9) | **15** | Range 1 to 5x a week<br>≤ 2x week (*n* = 3)<br>≥ 3x week (*n* = 5)<br>NR/U (*n* = 1) | **8** | Range 1 to 3x a week<br>≤ 2x week (*n* = 7)<br>≥ 3x week (*n* = 1)<br>NR/U (*n* = 1) | **8** |
| | **Duration of session(s)** | Range 15 to 70 minutes<br>≤ 30 min (*n* = 14)<br>> 30 min (*n* = 7)<br>NR/U (*n* = 1) | **20** | Range 15 to 60 minutes<br>≤ 30 min (*n* = 14)<br>> 30 min (*n* = 6)<br>NR/U (*n* = 3) | **19** | Range 30 to 60 minutes<br>≤ 30 min (*n* = 5)<br>> 30 min (*n* = 4)<br>NR/U (*n* = 0) | **9** | Range 15 to 30 minutes<br>≤ 30 min (*n* = 9)<br>> 30 min (*n* = 0)<br>NR/U (*n* = 0) | **9** |
| | **Strategies in applying stimuli** | Preference assessment (*n* = 1); non-directive approach (*n* = 8); internal session structure (*n* = 4); unpatterned stimuli (*n* = 0); no demands (*n* = 1); other (*n* = 4); NR/U (*n* = 8) | **13** | Preference assessment (*n* = 10); non-directive approach (*n* = 10); internal session structure (*n* = 10); unpatterned stimuli (*n* = 7); no demands (*n* = 6); other (*n* = 5); NR/U (*n* = 7) | **17** | Preference assessment (*n* = 0); non-directive approach (*n* = 5); internal session structure (*n* = 1); unpatterned stimuli (*n* = 0); no demands (*n* = 0); other (*n* = 1); NR/U (*n* = 3) | **6** | Preference assessment (*n* = 4); non-directive approach (*n* = 5); internal session structure (*n* = 3); unpatterned stimuli (*n* = 4); no demands (*n* = 4); other (*n* = 2); NR/U (*n* = 3) | **6** |
| **Multisensory environment** | **Physical aspects of the MSE** | Size 12 to 68m$^2$ (*n* = 9); white room (*n* = 5); padding (*n* = 5); no daylight (*n* = 4); NR/U (*n* = 8) | **13** | Size 9 to 11m$^2$ (*n* = 2); white room (*n* = 1); padding (*n* = 0); no daylight (*n* = 0); NR/U (*n* = 21) | **3** | Size 20 to 68m$^2$ (*n* = 4); white room (*n* = 3); padding (*n* = 1); no daylight (*n* = 1); NR/U (*n* = 4) | **5** | Size 9 to 32m$^2$ (*n* = 2); white room (*n* = 1); padding (*n* = 0); no daylight (*n* = 1); NR/U (*n* = 6) | **3** |
| | **Social aspects of the MSE** | Individual session (*n* = 13);<br>Group session (*n* = 6);<br>NR/U (*n* = 2) | **19** | Individual session (*n* = 17);<br>Group session (*n* = 0);<br>NR/U (*n* = 7) | **17** | Individual session (*n* = 3);<br>Group session (*n* = 5);<br>NR/U (*n* = 1) | **8** | Individual session (*n* = 8);<br>Group session (*n* = 1);<br>NR/U (*n* = 0) | **9** |
| **Support during snoezelen** | **Type of support person** | Therapist (*n* = 6); carer/ nurse (*n* = 5); teacher (*n* = 2); other (*n* = 5); NR/U (*n* = 5) | **16** | Therapist (*n* = 11); carer/ nurse (*n* = 7); teacher (*n* = 0); other (*n* = 2); NR/U (*n* = 7) | **17** | Therapist (*n* = 0); carer/ nurse (*n* = 5); teacher (*n* = 1); other (*n* = 0); NR/U (*n* = 3) | **6** | Therapist (*n* = 5); carer/ nurse (*n* = 2); teacher (*n* = 0); other (*n* = 1); NR/U (*n* = 3) | **6** |
| | **Training of support person** | Reported with (some) details (*n* = 2); reported without details (*n* = 2); not reported (*n* = 17) | **4** | Reported with (some) details (*n* = 3); reported without details (*n* = 7); not reported (*n* = 14) | **10** | Reported with (some) details (*n* = 0); reported without details (*n* = 2); not reported (*n* = 7) | **2** | Reported with (some) details (*n* = 1); reported without details (*n* = 4); not reported (*n* = 4) | **5** |
| | **Role of support person** | Active (*n* = 7); passive (*n* = 3); active and passive (*n* = 4); NR/U (*n* = 7) | **14** | Active (*n* = 7); passive (*n* = 1); active and passive (*n* = 1); NR/U (*n* = 15) | **9** | Active (*n* = 5); passive (*n* = 1); active and passive (*n* = 1); NR/U (*n* = 2) | **7** | Active (*n* = 4); passive (*n* = 0); active and passive (*n* = 1); NR/U (*n* = 4) | **5** |

*Note:* n *= number of articles; ID = intellectual disability; DM = dementia; MSE = multisensory environment; NR/U = not reported or unclear; (semi-)open queries excluded.*

## References

Cheng, S. C., Hsu, W. S., Shen, S. H., Hsu, M. C., & Lin, M. F. (2017). Dose-Response Relationships of Multisensory Intervention on Hospitalized Patients With Chronic Schizophrenia. *The journal of nursing research : JNR, 25*(1), 13-20. doi:10.1097/jnr.0000000000000154

Collier, L., Staal, J., & Homel, P. (2018). Multisensory environmental therapy (Snoezelen) for job stress reduction in mental health nurses: a randomized trial. *International Journal of Complementary & Alternative Medicine, 11*(1), 49-54.

Di Taranto, C., Procenesi, L., Paterniti, A. M., Loppi, E., Speranza, A. R., Siracusano, A., & Niolu, C. (2022). Outcome of the use of the Snoezelen Multisensory Room in an alexithymic patient: a case report. *Rivista di Psichiatria, 57*(3), 134-140. doi:10.1708/3814.37992

Gómez, C., Poza, J., Gutiérrez, M. T., Prada, E., Mendoza, N., & Hornero, R. (2016). Characterization of EEG patterns in brain-injured subjects and controls after a Snoezelen® intervention. *Computer methods and programs in biomedicine, 136*, 1-9. doi:10.1016/j.cmpb.2016.08.008

Hauck, Y., Rivers, C., & Doherty, K. (2008). Women's experiences of using a Snoezelen room during labour in Western Australia. *Midwifery, 24*(4), 460-470. doi:10.1016/j.midw.2007.03.007

Hotz, G. A., Castelblanco, A., Lara, I. M., Weiss, A. D., Duncan, R., & Kuluz, J. W. (2006). Snoezelen: A controlled multi-sensory stimulation therapy for children recovering from severe brain injury. *Brain Injury, 20*(8), 879-888. doi:10.1080/02699050600832635

Kim, M. K., & Park, N. K. (2022). Evaluating the Impact of a Multisensory Environment on Target Behaviors of Children With Autism Spectrum Disorder. *HERD, 15*(2), 163-179. doi:10.1177/19375867211050686

Lavie, E., Shapiro, M., & Julius, M. (2005). Hydrotherapy combined with Snoezelen multi-sensory therapy. *International Journal of Adolescent Medicine and Health, 17*(1), 83-87. doi:10.1515/IJAMH.2005.17.1.83

Lehrer, H., Dayan, I., Elkayam, K., Kfir, A., Bierman, U., Front, L., . . . Aidinoff, E. (2022). Responses to stimuli in the 'snoezelen' room in unresponsive wakefulness or in minimally responsive state. *Brain Injury, 36*(9), 1167-1175. doi:10.1080/02699052.2022.2110286

Leng, T. R., Woodward, M. J., Stokes, M. J., Swan, A. V., Wareing, L. A., & Baker, R. (2003). Effects of multisensory stimulation in people with Huntington's disease: A randomized controlled pilot study. *Clinical rehabilitation, 17*(1), 30-41. doi:10.1191/0269215503cr582oa

Manesh, M. J., Kalati, M., & Hosseini, F. (2015). Snoezelen room and childbirth outcome: A randomized clinical trial. *Iranian Red Crescent Medical Journal, 17*(5). doi:10.5812/ircmj.17(5)2015.18373

Momeni, M., Jamshidimanesh, M., & Ranjbar, H. (2020). Effectiveness of a snoezelen room on fear, anxiety, and satisfaction of nulliparous women: A randomized controlled trial. *Iranian Journal of Psychiatry and Behavioral Sciences, 14*(2). doi:10.5812/ijpbs.89168

Nelson, R., & Hummel, E. (2022). Using Multisensory Interventions to Address Anxiety, Agitation, and Pain in Adults Receiving Treatment for a Substance Use Disorder. *Therapeutic recreation journal, 56*(4), 522-539. doi:10.18666/TRJ-2022-V56-I4-11579

Nielsen, J. H., & Overgaard, C. (2020). Healing architecture and Snoezelen in delivery room design: a qualitative study of women's birth experiences and patient-centeredness of care. *BMC Pregnancy & Childbirth, 20*(1), 1-11. doi:10.1186/s12884-020-02983-z

Poza, J., Gómez, C., Gutiérrez, M. T., Mendoza, N., & Hornero, R. (2013). Effects of a multi-sensory environment on brain-injured patients: Assessment of spectral patterns. *Medical Engineering and Physics, 35*(3), 365-375. doi:10.1016/j.medengphy.2012.06.001

Priyadharsini, T., & Mukil Singh, M. E. (2016). Effects of snoezelen in the management of children with cerebral palsy who exhibits maladaptive behaviour in selected special care unit, Coimbatore, India. *International Journal of Research in Ayurveda and Pharmacy, 7*(3), 100-102. doi:10.7897/2277-4343.073121

Reddon, J. R., Hoang, T., Sehgal, S., & Marjanovic, Z. (2004). Immediate Effects of SnoezelenÂ® Treatment on Adult Psychiatric Patients and Community Controls. *Current Psychology: A Journal for Diverse Perspectives on Diverse Psychological Issues, 23*(3), 225-237. doi:10.1007/s12144-004-1022-1

Schofield, P. (2009). Snoezelen within a palliative care day setting: A randomized controlled trial investigating the potential. *International Journal on Disability and Human Development, 8*(1), 59-65.

Schofield, P., & Davis, B. (2000). Sensory stimulation (snoezelen) versus relaxation: A potential strategy for the management of chronic pain. *Disability and rehabilitation, 22*(15), 675-682. doi:10.1080/096382800445470

Schofield, P., Davis, B., & Hutchinson, R. (1998a). Evaluating the use of Snoezelen and chronic pain: the findings of an investigation into its use (Part II). *Complementary therapies in nursing & midwifery, 4*(5), 137-143. doi:10.1016/S1353-6117(98)80088-7

Schofield, P., Davis, B., & Hutchinson, R. (1998b). Snoezelen and chronic pain: developing a study to evaluate its use (Part I). *Complement Ther Nurs Midwifery, 4*(3), 66-72. doi:10.1016/s1353-6117(98)80057-7

Shapiro, M., Melmed, R. N., Sgan-Cohen, H. D., Eli, I., & Parush, S. (2007). Behavioural and physiological effect of dental environment sensory adaptation on children's dental anxiety. *European journal of oral sciences, 115*(6), 479-483. doi:10.1111/j.1600-0722.2007.00490.x

Unwin, K. L., Powell, G., & Jones, C. R. G. (2021). A sequential mixed-methods approach to exploring the experiences of practitioners who have worked in multi-sensory environments with autistic children. *Research in developmental disabilities, 118*. doi:10.1016/j.ridd.2021.104061

Unwin, K. L., Powell, G., & Jones, C. R. G. (2022). The use of Multi-Sensory Environments with autistic children: Exploring the effect of having control of sensory changes. *Autism, 26*(6), 1379-1394. doi:10.1177/13623613211050176

Yang, X. Y., Zhang, B. B., Zhao, L., & Chen, X. (2021). Effect of multi-sensory stimulation on children with global developmental delay. *Asian journal of surgery, 44*(10), 1308-1309. doi:10.1016/j.asjsur.2021.06.051
